# Structure and mechanism of bactericidal mammalian perforin-2, an ancient agent of innate immunity

**DOI:** 10.1126/sciadv.aax8286

**Published:** 2020-01-29

**Authors:** Tao Ni, Fang Jiao, Xiulian Yu, Saša Aden, Lucy Ginger, Sophie I. Williams, Fangfang Bai, Vojtěch Pražák, Dimple Karia, Phillip Stansfeld, Peijun Zhang, George Munson, Gregor Anderluh, Simon Scheuring, Robert J. C. Gilbert

**Affiliations:** 1 Division of Structural Biology, Wellcome Centre for Human Genetics, University of Oxford, Roosevelt Drive, Oxford OX3 7BN, UK.; 2 Department of Anesthesiology, Weill Cornell Medical College, 1300 York Ave., New York, NY 10065, USA.; 3 Calleva Research Centre for Evolution and Human Sciences, Magdalen College, University of Oxford, Oxford OX1 4AU, UK.; 4 Department of Molecular Biology and Nanobiotechnology, National Institute of Chemistry, Hajdrihova 19, 1000 Ljubljana, Slovenia.; 5 Department of Biochemistry, University of Oxford, South Parks Road, Oxford OX1 3QU, UK.; 6 Department of Microbiology and Immunology, University of Miami Miller School of Medicine, Miami, FL 33136, USA.; 7 Department of Physiology and Biophysics, Weill Cornell Medical College, 1300 York Ave., New York, NY 10065, USA.

## Abstract

Perforin-2 (MPEG1) is thought to enable the killing of invading microbes engulfed by macrophages and other phagocytes, forming pores in their membranes. Loss of perforin-2 renders individual phagocytes and whole organisms significantly more susceptible to bacterial pathogens. Here, we reveal the mechanism of perforin-2 activation and activity using atomic structures of pre-pore and pore assemblies, high-speed atomic force microscopy, and functional assays. Perforin-2 forms a pre-pore assembly in which its pore-forming domain points in the opposite direction to its membrane-targeting domain. Acidification then triggers pore formation, via a 180° conformational change. This novel and unexpected mechanism prevents premature bactericidal attack and may have played a key role in the evolution of all perforin family proteins.

## Teaser

Bactericidal perforin-2 assembles an inactivated pre-pore complex and undergoes a 180° reconfiguration for membrane attack.

## INTRODUCTION

Mammalian innate immunity is mediated by a range of factors including the membrane attack complex (MAC), which is deployed to kill invading microbes such as *Neisseria* species, while perforin-1 is used by cytotoxic lymphocytes to deliver granzymes into host cells targeted for destruction (*1*, *2*). Both the MAC complex and perforin-1 are known to form large β-barrel pores using similar mechanisms (*1*–*5*). Perforin-2 (PFN2) has recently been identified as a novel and critically important component of innate immunity conserved throughout the animal kingdom (*6*–*9*) and evolutionarily close to the last common ancestor of the MAC and perforin-1 (*7*, *10*). PFN2 is constitutively expressed in macrophages; however, its expression can be induced in many different cell types by interferons (α, β, and γ), bacterial infection, and pathogen-associated molecular patterns such as lipopolysaccharide (LPS) (*10*–*14*). Knockdown or deletion of PFN2 renders isolated cells and whole animals susceptible to infection, morbidity, and, in many cases, death (*10*, *12*, *13*). In human patients with pulmonary nontuberculous mycobacteria, four-point mutations of PFN2 have been identified (*15*), and macrophages expressing these mutant versions of PFN2 are unable to control *Salmonella typhimurium* or *Staphylococcus aureus* infection (*15*).

Because of the presence of a canonical MAC-perforin (MACPF) domain, PFN2 has been proposed to form functional pores in bacterial membranes, leading to bacterial death and control of infection (*10*–*13*, *16*). However, unlike MAC components and perforin-1, PFN2 is initially synthesized as a type I transmembrane protein and found associated with the membranes of intracellular vesicles in which its large ectodomain (consisting mainly of the MACPF domain and a unique P2 domain characteristic of PFN2) projects into the lumen. The PFN2 cytosolic tail, with a ubiquitin modification site, is then responsible for directing PFN2 to bacteria-encapsulating phagosomes (*13*) where it is thought to be further activated by proteolytic cleavage from its transmembrane anchor *(**12**)*. In this study, we provide a detailed framework for understanding the role of PFN2 in defense against intracellular bacteria, showing that it first assembles into a pre-pore state as a ring of 16 subunits, the pore-forming transmembrane hairpins (TMHs) of which point in the opposite direction to their membrane-binding domains. A complete (180°) reconfiguration of the MACPF and membrane-binding P2 domains triggered by acidification then results in pore formation. The evolutionary closeness of PFN2 to the common origin of all MACPF/cholesterol-dependent cytolysin (CDC) proteins suggests that this previously unobserved mechanism of pre-pore–to–pore transition may have played an important role in the evolutionary adaptation of the entire family to which it belongs.

## RESULTS

### Cryo-EM structure of the PFN2 pre-pore oligomer

**Fig. 1:**
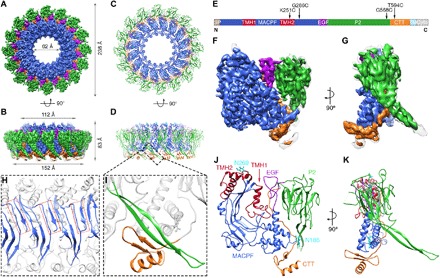
Cryo-EM structure of the PFN2 pre-pore oligomer. (**A** and **B**) Cryo-EM map of the mPFN2 pre-pore at 3.5 Å resolution as viewed from the top (A) and the side (B), color-coded by different domains (MACPF in blue, EGF in purple, P2 in green, and CTT in orange). (**C** and **D**) Top and side views of the atomic model of the mPFN2 pre-pore shown as ribbons; domains are colored as in (A). (**E**) Domain organization of the mPFN2: SP, signal peptide; TM, transmembrane helix; and Cyto, cytosolic tail. (**F** and **G**) Cryo-EM map of one subunit of the pre-pore colored as in (A). (**H**) The β sheet formed between MACPF domains marked with a dashed rectangular box. (**I**) Close-up view of the four-stranded β sheet formed between the CTT and the adjacent P2. (**J** and **K**) Ribbon representation of the pre-pore subunit as colored in (A); TMH-forming α helices are colored in red, and the conserved N-link glycans are colored in cyan.

**Fig. 2:**
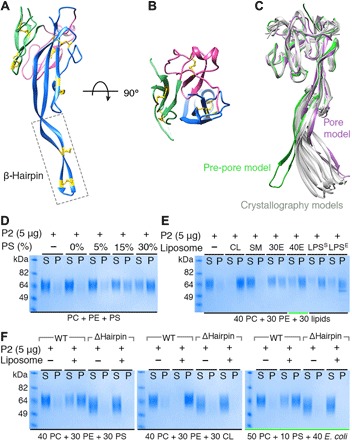
The P2 domain β hairpin is responsible for membrane binding. (**A** and **B**) Atomic structure of P2 domain showing pseudo-threefold symmetry repeat colored blue, pink, and green. Disulfide bonds linking the β strands are labeled in yellow; the β-hairpin region is marked with a dashed rectangular box. (**C**) Superimposition of the P2 cryo-EM structure (pre-pore model in green and pore model in purple) and 10 crystallographically distinct copies of the P2 domain (gray). (**D** and **E**) Ultracentrifugation-based liposome-binding assays of mPFN2 P2 domain; the liposomes contained different concentrations of phosphatidylserine (PS) (D) or negatively charged or neutral lipids (E). PC, phosphatidylcholine; PE, phosphatidylethanolamine; CL, cardiolipin; SM, sphingomyelin; 30E, 30% of *E. coli* total lipid extract; 40E, 40% of *E. coli* total lipid extract; LPS^S^, LPS from *S. enterica*; LPS^E^, LPS from *E. coli.* (**F**) Ultracentrifugation-based liposome-binding assays of mPFN2 P2 domain and P2Δhairpin truncation mutant. Green underlines in (E) and (F) indicate the same liposome composition with 50% PC/10% PS/40% *E. coli* total lipid extract. WT, wild type.

To characterize the basis for PFN2 activity, we have determined the molecular structure of its ectodomain in the context of a pre-pore assembly formed in solution at endosomal pH 5.5 (Fig. 1, A and B; fig. S1; and table S1). Most of the oligomers observed were full rings containing 16 subunits, with a very small percentage (<2%) of incomplete rings (arcs) with similar curvature to the ring (fig. S1, A and B), as observed with other MACPF/CDC family proteins including perforin-1 (*1*, *2*, *4*, *5*). We determined the near-atomic resolution structure of this pre-pore form at an overall resolution of 3.5 Å (fig. S1C), with local resolution varying from 3.3 to 4.75 Å (fig. S1D). The resolution of the peripheral P2 domain is relatively low in the cryo–electron microscopy (cryo-EM) structure, and in particular, an extended β-hairpin region was not well resolved. However, by determining a structure of the P2 domain in isolation by x-ray crystallography (at 2.05 Å resolution; Fig. 2, A and B, and table S2), we were able to build an atomic model for the whole complex (Fig. 1, C and D) into the cryo-EM map (Fig. 1, A and B). This revealed the presence of the expected MACPF family domain at the protein’s N terminus, followed by an epidermal growth factor (EGF)–like repeat and the P2 domain and finally a C-terminal tail (CTT) not present in other MACPF/CDC family proteins (Fig. 1, E to G).

As observed in other MACPF/CDC proteins, such as the Apicomplexan perforin–like protein *Tg*PLP1 (*17*) and the pneumolysin pre-pore (*18*, *19*), the MACPF domains make extensive complementary contacts with each other in the PFN2 pre-pore structure (Fig. 1H). However, the CTT region of PFN2 (A577-I642, colored orange in Fig. 1) threads back from the end of the globular region of the P2 domain, passes down between the MACPF and P2 domains, and folds into a two-stranded β sheet on the top of the MACPF domain before terminating in an α helix. This packs the CTT’s β strands against the β hairpin of the P2 domain from an adjacent subunit to create a continuous four-stranded intermolecular β sheet (Fig. 1, D and I). The folding of the PFN2 polypeptide chain from one subunit onto the next generates an additional stable intersubunit interface (Fig. 1D) absent in other MACPF/CDC superfamily members.

The pre-pore of PFN2 has a distinctive hat-like appearance in profile (Fig. 1, A to D) with the MACPF and P2 domains arranged concentrically. The larger MACPF domain is located inside, and the smaller P2 domains toward the outside of the ring of subunits, linked via a truncated EGF-like domain (Fig. 1, E to G). The PFN2 MACPF domain is closely related to the equivalent region of perforin-1, with two sets of transmembrane hairpin (TMH1 and TMH2) α helices prominently displayed available to form a β sheet traversing a targeted membrane on pore formation (Fig. 1, J and K). Like perforin-1, the TMH regions of PFN2 are similar to each other in length, though shorter (40 and 45 for TMH1 and TMH2 in PFN2 compared to 53 and 57 residues in perforin-1, respectively). The MACPF domain also contains conserved glycosylation sites (Fig. 1J), in equivalent positions to those found in the MAC component C9 and perforin-1. Construction of a structure-based phylogenetic tree for the PFN2 MACPF domain (fig. S2) places it at a position entirely consistent with sequence-based phylogenetic analyses, which indicated that perforin-1 derives from a PFN2 ancestral gene duplication (*7*, *9*).

### The β hairpin of the P2 domain directs membrane recognition

We determined structures of the P2 domain in isolation by x-ray crystallography in two crystal forms, with two crystallographically distinct copies in space group P2_1_ at 2.05 Å and eight copies in space group P1 at 3.17 Å (table S2). In both crystal forms, the extended β hairpin of the P2 domain could be unambiguously modeled and extends for 45 Å (Fig. 2, fig. S3, and table S2). The P2 domain has pseudo-threefold symmetry (Fig. 2, A and B, and fig. S3B) with a topological arrangement of constituent modules not previously observed (fig. S3A) distinct from the membrane-binding APCβ domains of Apicomplexan perforin–like proteins (*17*). Each repeat contains three strands with a conserved interstrand disulfide bond [pairwise root mean square deviation (RMSD) = 0.76 to 1.00 Å calculated from the three repeats]. The first repeat is much more extended than the other two and gives rise to the long β-hairpin projection, which is braced by two additional disulfide bonds, and enriched in positively charged residues along its length (fig. S3, B and C) and with mainly hydrophobic residues (LKIF) forming its tip (fig. S3B). Overlay of the cryo-EM and 10 crystallographically distinct copies of the isolated P2 domain structure (see above, Fig. 2C) indicates flexibility in the β hairpin. The pore-forming conformation of the P2 hairpin echoes the crystal structures (see below), whereas the pre-pore positioning of the hairpin is distorted away from its ensemble of resting (crystallographic) conformations (Fig. 2C). The rigidifying disulfide bonds and the hydrophobic residues in the P2 domain tip are highly conserved across species, although the identities of the residues along the β hairpin mostly are not (fig. S4, dashed black box). The highly basic nature of this projection (fig. S3D) and functional analogy to other family members including *Tg*PLP1, perforin-1, and the CDCs strongly suggest that the β hairpin will be involved in membrane targeting and binding by PFN2.

To test this hypothesis, we made use of liposome sedimentation experiments with the P2 domain alone (Fig. 2, D to F). Liposomes containing phosphatidylcholine (PC) and phosphatidylethanolamine (PE) showed no sedimentation of the P2 domain. When adding to these base lipids increasing concentrations of phosphatidylserine (PS) from 5 to 30%, the binding efficiency was gradually enhanced (Fig. 2D). Given that negatively charged lipids such as cardiolipin, *Escherichia coli* total lipid extracts, and LPS from *E. coli* and *Salmonella enterica* are characteristic of major bacterial membrane lipid species, we also tested and confirmed preferential binding of the P2 domain to these lipids (Fig. 2E). In contrast, the P2 domain showed almost no binding to liposomes containing sphingomyelin, which is a neutrally charged lipid found in animal cell membranes (Fig. 2E). Truncating the β hairpin (residues Y427-V452) within the P2 domain (P2Δhairpin mutant) abolished its binding to PS, cardiolipin, and *E. coli* lipids, confirming the role of this region in membrane binding by PFN2 (Fig. 2F). Although the CTT domain contributes directly to the intersubunit contacts with the P2 β hairpin and seems in the pre-pore assembly to be positioned such that it would lie along the top of the bilayer surface, truncation of the CTT domain (Δ606-652aa) within the ectodomain of mPFN2 showed no effect on the protein’s lipid binding activity (fig. S3E). Thus, membrane binding seems to be conferred exclusively by the tip region of the P2 domain β hairpin.

### Dynamics of PFN2 pre-pore assembly on membranes

**Fig. 3:**
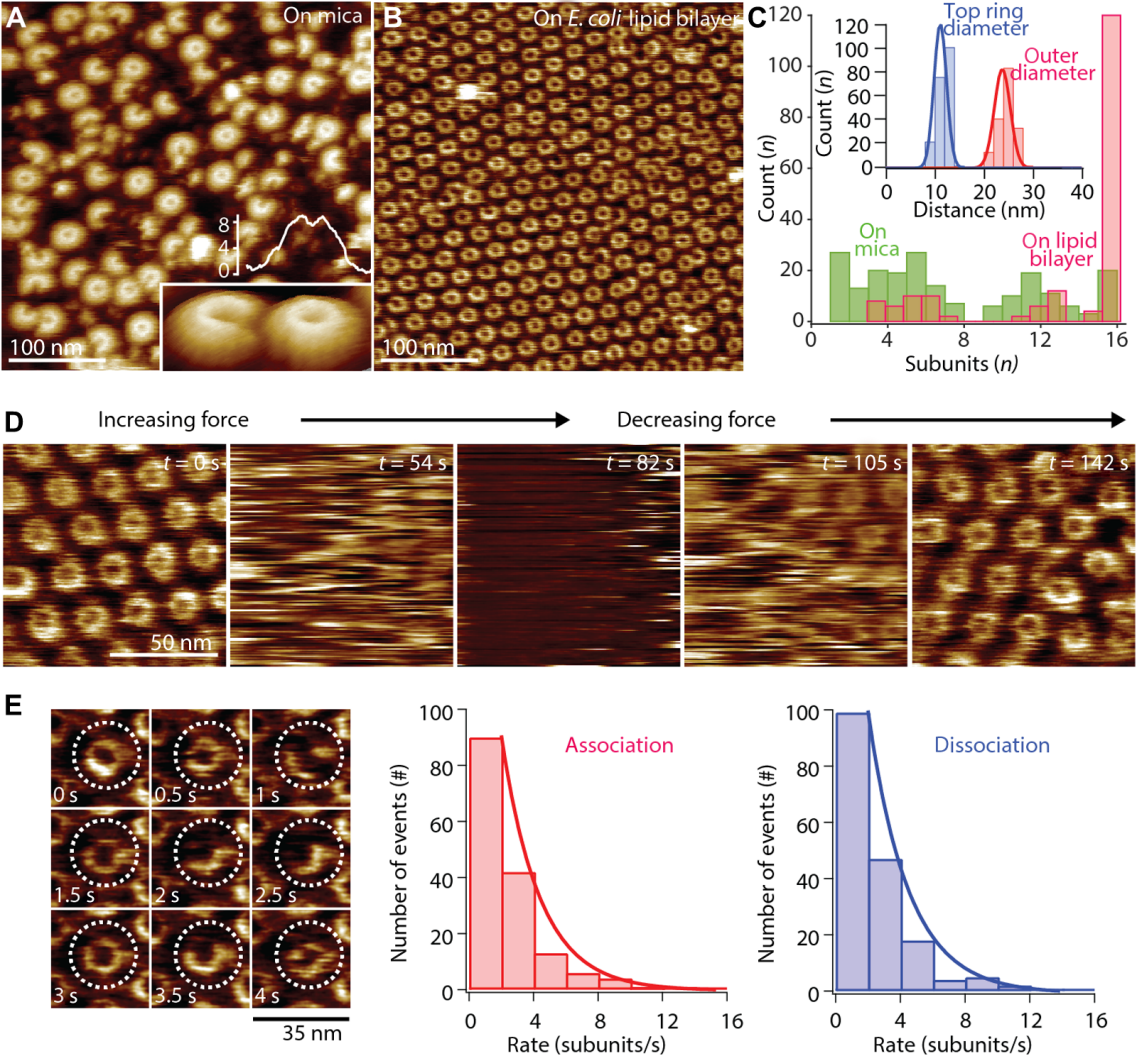
HS-AFM of MPFN2 pre-pore structure and assembly. (**A**) HS-AFM high-resolution image of mPFN2 on mica. Most of the mPFN2 oligomers are incomplete crescent-shaped assemblies, likely due to restricted lateral diffusion on mica necessary for full assembly of rings. The individual subunits can be distinguished (inset image). The inset section profile shows that pre-pore rings have a height of ~8.5 nm, which is consistent with the cryo-EM pre-pore model. (**B**) HS-AFM image of mPFN2 on an *E. coli* lipid bilayer. Essentially all mPFN2 oligomers are complete rings that assemble into a close-packed hexagonal lattice. (**C**) Histogram analysis of mPFN2 assembly on mica and on an *E. coli* lipid bilayer. On mica, mPFN2 displays a wide range of arc angles from one subunit to a full ring. On the lipid bilayer, ≥80% of mPFN2 molecules form complete ring assemblies, with a top ring diameter of 11.2 ± 0.9 nm and an outer diameter of 23.8 ± 1.2 nm (inset). (**D**) HS-AFM images of mPFN2 oligomers on an *E. coli* lipid bilayer (*t* = 0 s and *t* = 142 s) exposed to HS-AFM tip force induced removal (*t* = 54 s), giving access to an empty bilayer (*t* = 82 s) followed by assembly recovery (*t* = 105 s), in agreement with bilayer surface–associated pre-pores. (**E**) HS-AFM images (frame acquisition speed: 500 ms) and histogram analysis of mPFN2 subunit association (2.4 s^−1^) and dissociation (2.3 s^−1^) rates. Around 10 association/dissociation events were measured for identical mPFN2 oligomers in a single time frame, and more than 15 oligomers were recorded (total events, both >150). Histograms of association and dissociation rates were fit to a single exponential decay function. Full rings, where all subunits have neighbors on both sides, are stable over extended periods of time.

PFN2 pre-pore oligomeric assemblies were studied further using high-speed atomic force microscopy (HS-AFM) (Fig. 3). When PFN2 monomers were simply added directly to a freshly cleaved atomically flat mica surface (Fig. 3A and movie S1), HS-AFM found a wide distribution of arcs as well as rings of subunits with sizes ranging from 1 to 16 subunits (16 subunits corresponding to the complete ring) (Fig. 3C, green). In these experiments, only 7% of the assemblies overall formed complete rings (16 subunits). In contrast, on lipid bilayers (Fig. 3B), the overwhelming majority of membrane-bound oligomers formed complete rings (Fig. 3C, magenta). The complete rings were in total ≥80% of the assemblies found on lipid bilayers, uncompleted rings (arcs) only occurring on the competing borders of adjacent patches of hexagonal rings. While the assemblies displayed no long-range order on the mica, on the membrane, the rings arranged hexagonally (Fig. 3B). From this, we concluded that PFN2 existed as monomers or small-sized oligomers in solution and that their direct adsorption to the mica, where lateral diffusion is hindered, did not allow the protein to form complete rings. In contrast, when incubated on a membrane, where lateral two-dimensional (2D) diffusion is allowed, PFN2 formed stable rings 24 nm in diameter, which furthermore assembled into a hexagonal, i.e., the densest, molecular packing (Fig. 3C, inset, and movie S2). To further explore whether the oligomers observed using HS-AFM were membrane-bound PFN2 pre-pores, we performed a force-sweep experiment to clear them from the area of observation: The distribution of oligomeric assemblies recovered by diffusion within ~10 s after minimizing the force applied to the tip, as expected for membrane-associated structures (Fig. 3D and movie S3). The occasional observation of incomplete PFN2 rings on membranes allows the determination of subunit association and dissociation rates, which were similar: 2.4 subunits·s^−1^ and 2.3 subunits·s^−1^, respectively (Fig. 3E).

The dimensions of the pre-pores measured by AFM [~24 nm outer diameter with an upper protrusion (top ring) diameter of ~11 nm; Fig. 3C, inset] provide further evidence of the orientation of the pre-pore oligomer with respect to the membrane. Consulting the PFN2 pre-pore structure, an upper protrusion of ~11 nm diameter arises when the TMH regions of the MACPF domain are accessible to the HS-AFM tip, orienting the P2 domain β hairpin toward the lipid bilayer. If the oligomer were bound the other way up, then the diameter of the upper surface of the membrane-bound pre-pore would be ~15 nm. Thus, HS-AFM shows dynamic pre-pore formation as a lateral diffusion-dependent process leading to ring-shaped assemblies with ~24 nm outer diameter. The pre-pores can adhere to the bilayer via their β-hairpin exposing face, in agreement with the structural charge distribution of the pre-pore (fig. S3D) and lipid-binding experiments (Fig. 2). In this orientation, the membrane-inserting regions of the MACPF domain would point directly away from the targeted membrane.

### Acidic pH triggers PFN2 pre-pore–to–pore transition

**Fig. 4:**
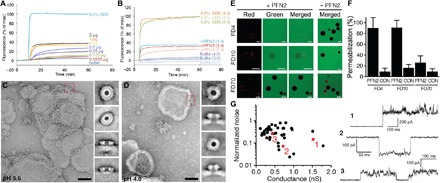
Pore-forming activity of mPFN2. Liposome leakage assay of purified mPFN2 protein incubated with liposomes containing 50% PC, 10% PS, and 40% *E. coli* total lipid extract. Liposome leakage was monitored by measuring fluorescence of released sulforhodamine B, and 0.2% SDS treatment was used as a positive control of 100% dye leakage. (**A**) Liposome leakage was dependent on mPFN2 concentration. (**B**) Increased sulforhodamine B release was observed with liposomes preformed at acidic pH (4.0 and 3.0) compared to at pH 5.5. Final leakage of sulforhodamine B is expressed as mean ± SD from three technical replicates, as shown in fig. S4 (B and C). All data shown are representative of three independent experiments. (**C**) Representative negative-stain micrograph and 2D class averages of mPFN2 incubated with liposomes containing 50% PC, 10% PS, and 40% *E. coli* total lipid extract at pH 5.5, with a dashed red box showing a side view of a membrane-bound pre-pore. Scale bar, 50 nm. (**D**) Representative negative-stain micrograph and 2D class averages of mPFN2 incubated with liposomes containing 40% PC, 30% PE, and 30% SM at pH 4.0; the lumen of the pores is much bigger than that of the pre-pores shown in (C). A dashed red box highlights a side view of a pore. Scale bar, 50 nm. (**E**) Permeabilization of GUVs composed of POPC:POPS:*E. coli* lipid extract (80:10:10; w/w) (+ rhodamine-DHPE; red channel) by addition of PFN2 (0.1 mg/ml) and fluorescent dextrans (FDs) at pH 5.7, after 60-min (FD4, 4 kDa) or 120-min (FD10 and FD70, 10 and 70 kDa) incubation. Scale bar, 20 μm. (**F**) Quantification of GUV permeabilization from conditions shown and described in (E). CON denotes control sample without addition of PFN2. (**G**) Pore formation of PFN2 on planar lipid bilayers composed of POPC:POPS:*E. coli* lipid extract (70:10:20; w/w) at 10 mM MES and 500 mM NaCl (pH 5.5). The red numbers on the graph correspond to the three traces shown (right). The loss of pore conductance in traces 2 and 3 is likely due to the loss of pore structures from membranes.

We next examined the membrane-perforating activity of mPFN2 using several complementary approaches. First, a sulforhodamine B–based leakage assay revealed PFN2 concentration–dependent dye leakage from pre-formed liposomes (PC/PS/*E. coli* total lipid extracts, 5:1:4) at pH 5.5, whereas no dye leakage was observed at pH 7.5 (Fig. 4A and fig. S5, A and B). At acidic pH 4.0 and 3.0, mPFN2 showed increased dye leakage compared to that at pH 5.5 (Fig. 4B and fig. S5C). Furthermore, negative-stain electron microscopy revealed that most of the membrane-bound mPFN2 oligomers were pre-pores with occasional pores at pH 5.5 (Fig. 4C and figs. S5D, red box, and S6A). Side views of these pre-pores displayed similar profiles to those of the cryo-EM structure, confirming that PFN2 adheres to the lipid bilayer via its P2 β hairpin and with the membrane-inserting regions of the MACPF domain facing in the pre-pore in the opposite direction (Figs. 1 and 4C). In contrast, multiple mPFN2 pores were formed on liposomes containing 40% *E. coli* total extract at pH 4.0, resulting in the clustering of the liposomes so that it was not easy to identify the individual mPFN2 oligomers (fig. S6C). Thus, we incubated mPFN2 with liposomes containing sphigomyelin (fig. S6, B and D), which showed much less binding with the P2 domain. The distributed pore-forming mPFN2 oligomers on the liposome at pH4.0 showed an enlarged lumen compared to that of the pre-pore (Fig. 4D, compared to C, and fig. S6D). Side views of membrane-inserted pores were very different from those of pre-pores, with obvious transmembrane channels and standing significantly taller than the pre-pore form (110 Å compared to 85 Å) (Fig. 4D).

Third, giant unilamellar vesicle (GUV) imaging in the presence of fluorescent dextrans (FDs) of increasing sizes showed that the PFN2 ectodomain forms pores, allowing almost complete equilibration of 4-kDa dextran (~2.8 nm diameter) and 10-kDa dextran (~4.6-nm diameter) and no leakage of 70-kDa dextran (~12 nm diameter) into the GUV interior (Fig. 4, E and F). The size-dependent passage of FDs through PFN2 pores matches what was previously observed with similar experiments on perforin-1, which forms oligomers of similar size to PFN2 (*4*). Fourth, channel conductance measurements revealed the formation of discrete pores in membranes targeted by PFN2 with a range of sizes and conductance noise—in line with observations made for perforin-1 pores (*4*)—but with a consistent pattern of pores opening in discrete steps due to a defined pre-pore–to–pore transition (Fig. 4G).

**Fig. 5:**
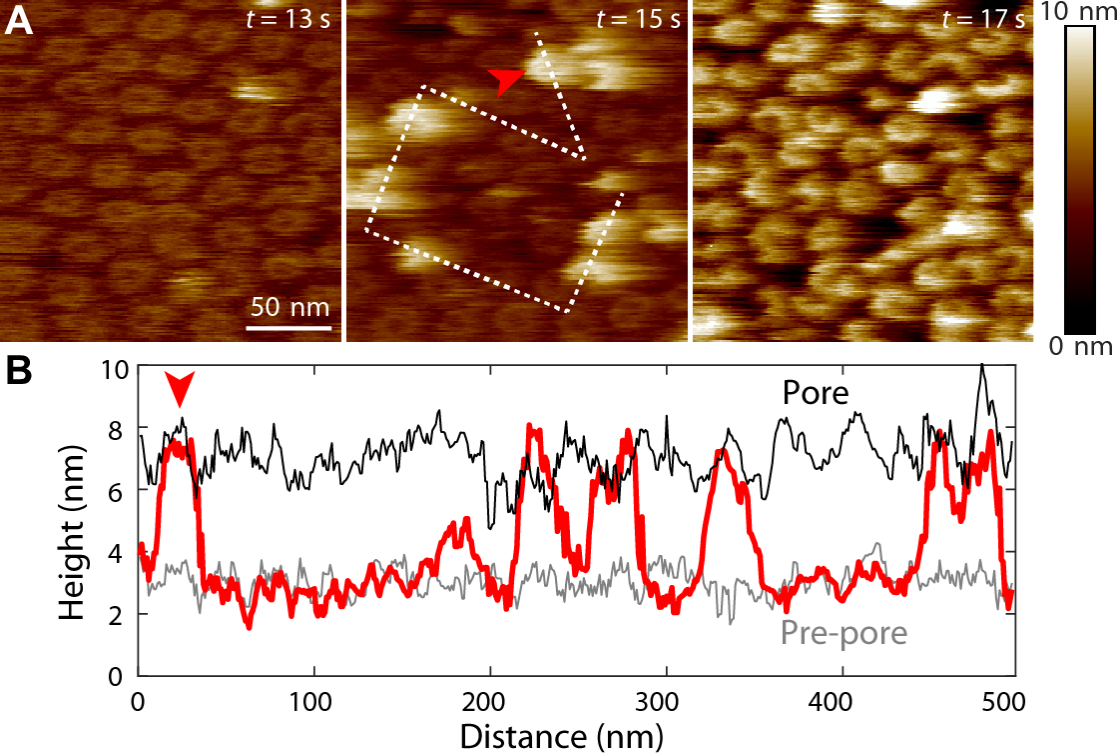
Pre-pore–to–pore transition observed by HS-AFM. (**A**) HS-AFM image (left) of mPFN2 on an *E. coli* lipid bilayer as in Fig. 3B then (middle and right) images of the same area as the pH is reduced to 3.6 to 4.0. An increase in oligomeric height results in a sharpening of the AFM image as the oligomers come more closely into contact with the cantilever tip. (**B**) Height profile of the transit shown with a white dashed line in (A) (middle), confirming an increase in oligomeric height above the targeted lipid bilayer on acid-triggered pore formation. The gray, red, and black lines indicate the height profile of pre-pore, mid-transition, and pore states, individually, along the path defined by the dashed line in (A). The red arrowheads in (A) and (B) show the same position.

Last, we performed direct pre-pore–to–pore activation experiments via HS-AFM when coupled to a buffer exchange system (*20*). Upon exposure to a low pH shock, the PFN2 pre-pore rings began to form pores after 14 s and all ~70 being imaged transited to the pore state within 3 s (Fig. 5 and movie S4). The resulting structures were higher (raised by ~4 nm) compared to the pre-pore state, in agreement with the pores imaged by negative-stain electron microscopy (Fig. 4D).

Overall, these results show that acidic pH triggers pre-pore–to–pore transformation, which is achieved by a 180° rotation of the MACPF and P2 domains with respect to each other so that membrane insertion can occur. This would require at least two significant conformational rearrangements: Rotation at the MACPF/P2 domain interface and unfurling of the TMHs into β hairpins. To test this hypothesis, we made use of two PFN2 disulfide bond mutants, following a strategy successfully implemented with perforin-1 and other MACPF/CDC family members (*2*). PFN2^G568C/T594C^ locks the CTT to the P2 domain, and PFN2^K251C/G286C^ prevents the deployment of the TMH2 region from the MACPF domain (fig. S7, A to C): Neither PFN2^G568C/T594C^ nor PFN2^K251C/G286C^ was active to form pores on liposomes when the engineered disulfide bridge was present at pH 5.5 (fig. S7, D to F), although pre-pore assembly was observed in both cases as with the wild-type (WT) protein (Fig. 4C and fig. S7, D and E). Addition of reducing reagent activated both mutants by cleaving the disulfide bond, allowing detachment of the MACPF domain from the P2 domain and the unfurling of the TMH2 region: 1 mM dithiothreitol (DTT) reduced PFN2^K251C/G286C^, but 10 mM DTT was necessary for the same effect on PFN2^G568C/T594C^, most likely because these cysteines are buried in the MACPF domain/P2 domain interface (fig. S7G). Together, these results demonstrate the need to release the interdomain lock between the MACPF domain and P2 domain to allow a 180° rotation, and then the unfurling of the MACPF domain TMHs, for pore formation to occur.

### Cryo-EM structure of the PFN2 pore

**Fig. 6:**
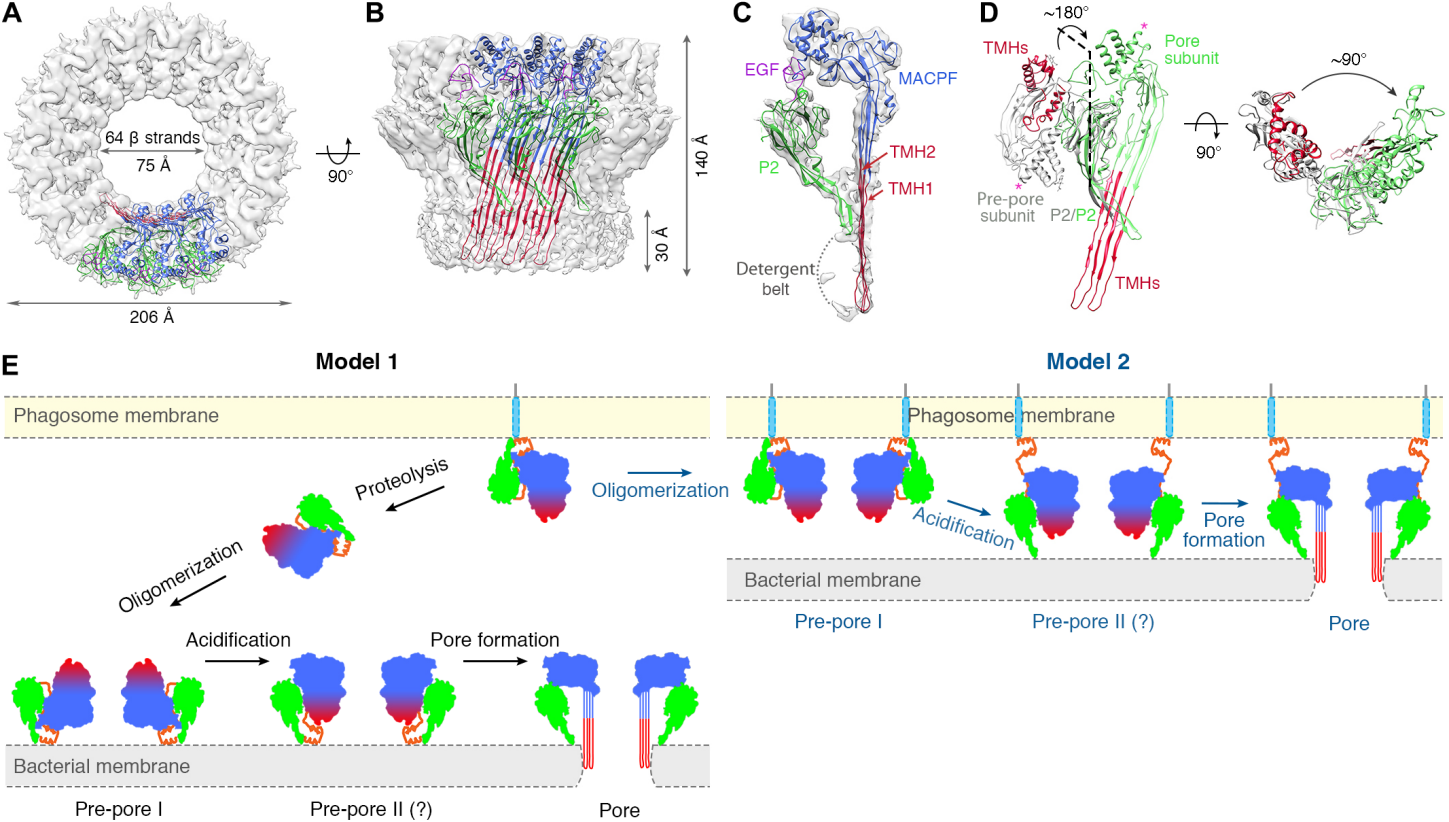
Cryo-EM structure of mPFN2 pore. (**A** and **B**) Top view (A) and side view (B) of mPFN2 model fitted in the cryo-EM map. (**C**) A single subunit segment of mPFN2 pore map (gray) with model fitted into the density viewed from the side. Domains were color-coded as in Fig. 1. (**D**) Superposition of mPFN2 monomer in the pre-pore form (gray) and the pore conformation (green), showing a 180° rotation of the MACPF domain viewed from the side and a 90° rotation from the above. The two TMHs were highlighted in red in both conformations, and the magenta asterisks indicate the same region in the two conformational states. (**E**) Two proposed models illustrating the conformational changes during the pre-pore–to–pore transition of mPFN2. In model 1, mPFN2 is released to the phagosome lumen after the cleavage from its transmembrane domain, and it assembles as a pre-pore at pH 5.5 on the bacterial membrane (Pre-pore I); further acidification (pH 4.0) of the phagosome triggers a ~180° reorientation of the MACPF domain leading to the two TMHs (red) facing the membrane (Pre-pore II), and then the helical TMH regions unfold to form a β-stranded barrel, which inserts into the membrane in pore formation (Pore). In model 2, mPFN2 assembles as a pre-pore on the phagosome membrane (at pH 5.5) while still tethered on the phagosome membrane via its transmembrane region; further acidification (pH 4.0) triggers a ~180° reorientation of the P2 domain, resulting in binding to a nearby bacterium (Pre-pore II) and subsequent pore formation in its surface membrane (Pore). Domains of mPFN2 in the schematic were color-coded as in Fig. 1: TMHs colored in red within the MACPF domain (blue), P2 in green, and CTT in orange; the transmembrane domain is shown as a light blue stick, and the cytosolic tail is shown as a gray line. For the pore conformation, only MACPF and P2 domains are shown, because the CTT domain is not resolved in the pore structure.

To generate a sample of pores for structure determination, we prepared pre-pores at pH 5.5 as for the structure reported above and then lowered the pH to 3.6-4.0 in the presence of Cymal-6 (fig. S8, A and B). The cryo-EM structure of the pore was determined to an overall resolution of ~5 Å (Fig. 6, A and B; fig. S8, C and D; and table S1). The resolution for the central MACPF region of the cryo-EM map is ~4.5 Å, whereas the peripheral regions have lower resolutions ranging from 5.0 to 9.2 Å (fig. S8, C and D). We were able to construct a model of the pore fitted to its cryo-EM density guided by the distinctive features of each PFN2 domain and their connectivity and the conserved two glycans visible in both structures. We were unable to resolve the CTT domain due to the absence of density for the CTT in the PFN2 pore map, suggesting that it may have become intrinsically disordered on pore formation. Our structure confirms the rotation between the MACPF and P2 domains by 180° and reveals other conformational changes accompanying membrane insertion including the deployment of TMH1 and TMH2 to generate a 64-stranded β-barrel (Fig. 6, B to D). In the pore form, the MACPF domain has rotated with respect to the P2 domain by ~180° when viewed from the side and by ~90° when viewed from above (looking down toward the membrane surface) compared to its position in the pre-pore, resulting in a compound screw rotation (Fig. 6D). The pore-forming oligomer structure is entirely consistent with the pores imaged directly on liposome membranes and is 206 Å in diameter and 140 Å tall from the top of the MACPF domain to the tip of the β-barrel (Fig. 6, A and B) (*1*, *2*, *5*, *21*, *22*).

## DISCUSSION

Our structures of mPFN2 pre-pore and pore forms provide intriguing insights into its mechanism of pore formation. Depending on whether PFN2 is released to the phagosome lumen by proteolysis *(**12**)*, two models can be proposed for the mechanism underlining pore formation by PFN2 (Fig. 6E). In model 1, monomeric PFN2 is released into the phagosome lumen after its cleavage from the phagosome membrane, binding to a target bacterial surface and oligomerizing upon it into a pre-pore assembly. Upon activation by acidification of the phagosome, the MACPF domain rotates by 180° with respect to the membrane-bound P2 domain, followed by the unfurling of its TMHs (Fig. 6E, left, model 1). If considered as a rigid body movement, the steric clash between MACPF domains will not allow rings of subunits to make this transition intact, and we hypothesize that pore formation begins with the opening of the pre-pore oligomer between two subunits, allowing the conversion to the pore state to begin. In an alternative model (Fig. 6E, right, model 2), PFN2 remains membrane-anchored in its pre-pore form and close approach of the phagosome membrane and bacterium is essential for the binding of the pre-pore to the bacterial surface via a 180° rotation of the P2 domain. Subsequently, the MACPF domain TMHs would be unfurled and inserted into the bacterial surface; in this model, the CTT would act as an interdomain lock by interacting with an adjacent subunit’s P2 domain β hairpin, as in the pre-pore structure. But upon pore formation on a bacterial membrane, the CTT might then become a flexible linker between the phagosome and bacterial membranes. However, in this model, the oligomerization of PFN2 into a pre-pore occurs before membrane binding, whereas in all other MACPF/CDC proteins membrane binding precedes oligomerization. In addition, rotation of the P2 domain with respect to the MACPF, as in model 2, also results in a clash between subunits. Which model outlined is more correct—i.e., whether the P2 domain binds the target membrane before or after pre-pore formation—and the exact conformational pathway taken in each case will require further experimentation and, most likely, a combination of high-resolution real-time AFM imaging with structural characterization of pore formation intermediates.

## CONCLUSION

Together, we have used cryo-EM to determine structures for the pre-pore and pore forms of PFN2, x-ray crystallography to determine multiple conformations of its membrane-binding P2 domain, high-speed AFM to image pre-pore and pore formation in real time, functional assays of PFN2 based on fluorescence imaging and spectroscopy and conductance measurements, and site-directed mutagenesis to test hypotheses arising from the structural and dynamic data. The outstanding insight from our study is that the TMH membrane-inserting regions of the pore-forming MACPF domain are oriented in the opposite direction to the membrane-binding region of the P2 domain in the PFN2 pre-pore. In all other pore-forming MACPF/CDC proteins, the membrane-binding domain and TMHs are oriented in the same direction in their membrane-bound pre-pore states (*23*, *24*). Domain reorientation during pore formation is not unique to the PFN2 transition: In the aerolysin pore-forming protein family, the pore-forming domain refolds inward on pore formation (*25*, *26*). But by contrast—and uniquely for MACPF/CDC proteins studied in molecular detail—PFN2 wholly reorients the relationship between its pore-forming domain and membrane-binding domains via a circumferential swing before deployment of its membrane-inserting TMHs. This arrangement may enable stringent control of pore formation such that formation occurs only upon phagosome acidification, for more effective killing of engulfed bacteria. We suggest that the three domains involved—the membrane-bound P2 domain, the EGF module at the hinge point, and the pore-forming MACPF domain—are specifically selected to enable this transition. The P2 domain helps to stabilize the pre-pore state by swapping between monomers and enables PFN2 to bind its targeted membrane. The EGF module provides a focal point for the rotation to occur, and the MACPF domain is adapted for the well-established helix-to-sheet transition accompanying MACPF/CDC pore formation (*27*) and for packing stably into oligomers in the two different orientations found in the pre-pore and pore.

The other domains out of which PFN2 is built are found in similar forms in many other MACPF/CDC family proteins, such as perforin-1 and components of the complement MAC as well as non–pore-forming family members like astrotactin-2 (*3*, *28*–*30*), and a related set of domains is found in the bacterial CDCs (*31*). So why are the domains arranged with the MACPF and P2 domains pointing in opposite directions in PFN2? Given the pH activation of pore formation, we suggest that this pre-pore arrangement holds PFN2 in an inactive state until the correct endosomal pH is achieved to facilitate effective killing of bacteria by membrane perforation. Given the antiquity of the PFN2 gene [see above and (*7*, *9*)], we further suggest that the configuration of all other MACPF/CDC proteins described with pore-forming and membrane-binding regions pointing in the same direction in the pre-pore state is an adaptation of the opposite configuration found in PFN2. This may have been facilitated by the loss of the CTT from ancestral PFN2 on duplication to initiate the evolution of perforin-1, because it is not found in other family members.

## MATERIALS AND METHODS

### Construct preparation, protein expression, and purification

The P2 domain of murine PFN2 (mPFN2; UniProt: A1L314) was cloned into the pHLsec vector (*32*) with Age I and Kpn I restriction cutting sites, resulting in a signal peptide in the N terminus of the proteins and a KTHHHHH tag in the C terminus. The signal peptide was cleaved during protein secretion into the medium, leaving an additional three residues Glu-Thr-Gly at the N terminus of the proteins. Full-length mPFN2 ectodomain (residues 20 to 652) was cloned into pHLsec-1D4, replacing the His6 tag with a rhodopsin 1D4 tag (*33*). Overlapping polymerase chain reaction was performed to introduce site-directed mutagenesis.

All the proteins in this study were produced recombinantly from mammalian human embryonic kidney (HEK) 293T cells or HEK293S cells using a transient transfection and expression protocol as described (*17*, *30*). To express the P2 domain in HEK293T cells for crystallization, 6 mg of purified DNA vector was transfected into 3 liters of HEK293T cells in the presence of the glycosylation inhibitor kifunensine (final concentration, 5 μM) with 12 mg of polyethylenimine. The transfected cells were harvested 4 to 5 days after transfection. Three liters of medium containing secreted proteins was harvested by centrifugation at 5000*g* for 45 min to remove the cell debris, filtered through a 0.22-μm filtration membrane, and dialyzed against 10× volume of phosphate-buffered saline [10 mM phosphate (pH 7.5) and 300 mM NaCl] overnight at 4°C. The dialyzed medium was supplemented with 20 mM imidazole and loaded onto a HisTrap HP (GE Healthcare) column overnight at room temperature and eluted with a linear imidazole gradient (20 to 500 mM imidazole) in 25 mM tris (pH 7.5) and 500 mM NaCl. The eluted proteins were pooled together and deglycosylated with Endo F1 overnight at 4°C, concentrated, and applied to a size-exclusion chromatography column (Superdex 75 16/600), and a peak corresponding to the monomeric species was concentrated to about 12 mg/ml in 10 mM Hepes (pH 7.5) and 150 mM NaCl. The proteins were flash-frozen in liquid nitrogen and stored in −80°C until further use.

Ectodomain of mPFN2 was purified from the medium using anti-1D4 antibody–coupled agarose resin. Six liters of mammalian expression medium with secreted mPFN2 was concentrated to 500 ml using a QuikStand device (Watson, Marlow) with a 10-kDa cutoff membrane, filtered, and incubated with anti-1D4 monoclonal antibody–coated resin preequilibrated in 25 mM tris (pH 7.5) and 500 mM NaCl buffer overnight at 4°C. The agarose resin with mPFN2 bound was then washed with 25 mM tris (pH 7.5) and 500 mM NaCl buffer before being eluted with 2 ml of 1D4 peptide at a concentration of 0.5 mg/ml. The sample was concentrated using a 30-kDa centrifugal concentrator (Millipore) to about 1.2 mg/ml and flash-frozen in liquid nitrogen for storage at −80°C. The mutants of the ectodomain were purified in the same way.

### Cryo-EM sample preparation and data collection

The mPFN2 ectodomain was incubated with buffer containing 50 mM citric acid buffer (pH 5.5) and 150 mM NaCl at 0.5 mg/ml at 37°C overnight to form pre-pore oligomers, which were further purified through size-exclusion chromatography (Superose 6 10/300). Fractions corresponding to the homogeneous oligomers were pooled together and concentrated to ~0.5 mg/ml. Tween 20 was added to the protein solution at a final concentration of 0.01% and incubated for 30 min before cryo-EM grids were prepared. The mPFN2 pore sample was prepared by lowering the pH of the pre-pore samples to pH 3.6 to 4.0 in the presence of 0.056% Cymal-6 (final concentration) at room temperature for 30 min. The pH was estimated by large-scale mixing of the pre-pore assembly buffer [50 mM citric acid (pH5.5) and 150 mM NaCl] with 1 to 1.5× volume of 170 mM citric acid (pH3.3) and 150 mM NaCl.

For cryo-EM, 3 μl of mPFN2 pre-pore (~0.3 mg/ml) was applied to a glow-discharged lacy carbon grid with ultrathin carbon support film (Agar, UK), blotted for 3.5 s in 100% humidity at 22°C, and flash-frozen in liquid ethane with an FEI Vitrobot Mark IV. The mPFN2 pore (~0.12 mg/ml) was frozen in the same way. The mPFN2 pre-pore grids were imaged using an FEI Polara electron microscope operating at 300 kV with semi-automated image acquisition via SerialEM. Micrographs were acquired with a Gatan K2 Summit direct electron detection camera operated in the counting mode at a calibrated magnification of ×48,076 and defocus values ranging from −1.0 to −2.5 μm. The mPFN2 pore data were collected using a Thermo Scientific Glacios operating at 200 kV with a Falcon III camera in linear mode. The details of data collection parameters are summarized in table S1.

### Cryo-EM image processing

For cryo-EM data of mPFN2 pre-pore, beam-induced motion correction was performed using MotionCor2 (*34*) to generate dose-weighted micrographs from all frames. The contrast transfer function was estimated using Gctf (*35*). XMIPP (*36*) was used for automatic particle picking, and the particles were further manually inspected. In total, 246,755 particles were extracted and initially binned 2×. After several rounds of reference-free 2D classification in RELION (*37*), good class averages representing the C16 symmetry were selected and further processed. About 5000 particles were used to generate an ab initio model in cryoSPARC (*38*), and the resulting initial model was used for 3D classification in RELION with C16 symmetry imposed. Particles from two classes (41,693 particles) with similar density maps were selected and reextracted at full size for further 3D refinement, resulting in a map with overall resolution of ~3.5 Å. The overall B factor was automatically determined using the RELION PostProcess tool, with an estimated B-factor applied of −144 Å^2^. The global resolution was estimated by the gold-standard Fourier shell correlation (FSC) = 0.143 criterion, with a soft mask applied around the protein density. The local resolution was estimated using RELION and presented in Chimera (*39*).

The dataset of mPFN2 pores was processed with similar procedures. In brief, automated particle picking was performed using Gautomatch (www.mrc-lmb.cam. ac.uk/kzhang/Gautomatch). A total of 44,046 particles contained in good 2D classes of all side and top views of C16 symmetry pores were selected after three rounds of 2D classification in RELION. An ab initio model generated with these particles in cryoSPARC was used for 3D classification in RELION without applying symmetry. The best class (24,936 particles) was further refined with C16 symmetry in RELION, resulting in a map with local resolution ranging from 4.1 to 9.2 Å. All subsequent post-processing steps were performed in the same way as described above for the pre-pore dataset.

### Model building into the cryo-EM reconstruction, refinement, and structure visualization

Phyre2 (*40*) was used to generate a homology model of the MACPF domain of mPFN2, using the perforin-1 crystal structure as the template [Protein Data Bank (PDB) entry code: 3NSJ]. The MACPF homology model was first fitted into the density map and manually rebuilt. The P2 domain crystal structure was manually fitted into the map, with major adjustment in the extended β hairpin. The remaining density map was manually traced. Real-space refinement was performed in Coot (*41*) and phenix.real_space_refine (*42*) using the B-factor sharpened density map. Secondary structure restraints from the x-ray crystallography model were applied when fitting the P2 domain into the cryo-EM map. A region of density with a local resolution of ~5 Å was modeled as a helix (residues L633-H643), which was also predicted from its secondary structure analysis. The presented map was filtered by its local resolution. The figures were prepared using Chimera (*39*) and PyMOL (The PyMOL Molecular Graphics System version 2.0, Schrödinger LLC).

To generate a model of the PFN2 pore, we first fitted the MACPF-EGF region (residues 31 to 371) minus TMH1 and TMH2 into the equivalent region of the pore map using Chimera (*39*). This also allowed us to confirm the correct hand for the map. We used the program iMODFIT (*43*) to then refine the MACPF-EGF domain structure into the density, whereby normal modes analysis is used to facilitate flexible fitting. This led to a straightening of the MACPF domain central β sheet in line with the deployment of the TMH1 and TMH2 regions. We then fitted the P2 domain, making use of all 11 structures to hand (10 crystals and 1 cryo-EM from the pre-pore). We found that the best fit was for chain B of the P1 crystal form and so used that in rigid body fitting into its density within the pore map. This process placed the C terminus of the EGF and the N terminus of the P2 domain close enough in space that the connectivity of the model could be restored manually in Coot (*41*). A further round of iMODFIT refinement was followed by the manual construction of two β hairpins for TMH1 and TMH2 using Coot (*41*) constrained by the alignment of residue Asn^269^ to it attached glycan density, followed by construction of a full 16-mer pore and refinement using phenix.real_space_refine (*42*) in isolation and incorporated in the Namdinator online server (*44*, *45*), until convergence was achieved. Namdinator runs made use of implicit solvent in the latter cycles of refinement.

### Structural phylogeny construction

Superimposition of homologous protein structures was performed using SHP (*46*) as previously reported (*47*). The phylogenetic tree was calculated using a pairwise evolutionary distance matrix determined from the superimposed domains. The tree representation was generated using the programs FITCH and DRAWTREE as part of the PHYLIP package (*48*).

### Protein crystallization, x-ray diffraction data collection, and structure determination

Crystallization screening was carried out by sitting-drop vapor-diffusion methods in CrystalQuick 96-well plates by mixing 100 nl of protein solution with 100 nl of reservoir and equilibrating against 95 μl of reservoir at 20°C. Deglycosylated P2 domain was concentrated to 10 to 15 mg/ml and crystallized in 0.1 M MES (pH 6.0) and 10% PEG-6000 (polyethylene glycol, molecular weight 6000) with 50 nl of additives (0.02 M l-cysteine stock). The crystals appeared after 2 weeks and grew to full size within 1 month. The crystals were harvested with 25% glycerol as the cryoprotectant in liquid nitrogen. The crystal diffraction data were collected on I24 and I03 beamlines at Diamond Light Source (Didcot, UK). For experimental phasing, the crystals were soaked with concentrated gold cyanide for 1 hour. The derivative dataset was collected at a wavelength of 1.04 Å (see table S2 for details of data collection).

A gold-derivative single anomalous dispersion (Au-SAD) analysis was first performed in HKL2map (*49*) to identify the heavy atom positions. The resulting heavy-atom coordinates were subsequently used together with a native dataset to carry out single isomorphous replacement anomalous scattering analysis in phenix.autosol (*50*). The resulting electron density was used in phenix.autobuild (*51*) to generate a partial model. The subsequent model building and refinement were carried out in Coot (*41*) and phenix.refine (*42*). The final refined model contains residues from Phe^376^ to Ile^579^, with one missing loop between Val^538^ and Pro^549^. The other crystal form was phased by molecular replacement using the refined P2 domain model with β-hairpin truncation. The electron density representing the β hairpin was clearly visible after rigid-body refinement. The subsequent model building and refinement were carried out in Coot and phenix.refine (*41*, *42*).

### Negative-stain electron microscopy

Wild-type PFN2 ectodomain and the disulfide-linked mutants were incubated with liposomes at room temperature for 30 min. A 3-μl mixture was applied to a glow-discharged copper grid covered with carbon film (Electron Microscopy Sciences), washed twice with dH_2_O, stained with 0.75% uranyl formate or 2% uranyl acetate for 1 min, and blotted dry with filter paper. Negative-stained images were collected using FEI Tecnai T12 microscopes at a nominal magnification of ×67,000, with a calibrated pixel size of 1.7 Å. The particles were manually picked and extracted in EMAN2 (*52*), and the subsequent image analysis was carried out with Imagic (*53*).

### Liposome sedimentation assay and sulforhodamine B liposome leakage assay

POPC (1-palmitoyl-2-oleoyl-*sn*-glycero-3-phosphocholine), POPS (1-palmitoyl-2-oleoyl-*sn*-glycero-3-phosphoserine), POPE (1-palmitoyl-2-oleoyl-*sn*-glycero-3-phosphoethanolamine), cardiolipin, sphingomyelin (brain, Porcine), and *E. coli* total lipid extract were purchased from Avanti Polar Lipids Inc. LPSs from *S. enterica* (L1887) and *E. coli* (L2018) were purchased from Sigma. Liposomes with the compositions indicated were prepared following standard protocols. Briefly, a total of 1 mg of lipids dissolved in chloroform was mixed and dried in a clean Pyrex tube overnight by desiccation in a desiccator attached to a VARIO-SP diaphragm pump (Vacuubrand). The lipid film was subsequently hydrated by adding 0.5 ml of solubilization buffer [20 mM Hepes (pH 7.5) and 150 mM NaCl] followed by vigorous vortexing and 5 to 10 freeze-thaw cycles, and the resulting mixture was then extruded through 100-nm polycarbonate membrane (Whatman) 11 times using an extruder (Avanti Polar Lipids Inc.). To prepare liposomes with LPSs incorporated, LPS dissolved in buffer [20 mM Hepes (pH 7.5) and 150 mM NaCl] was added into the solubilization buffer before freeze-thaw cycles and extrusion. The liposomes were stored at 4°C and used within 2 days. To perform liposome sedimentation assays, 5 μg of mPFN2 protein (P2 domain; P2Δhairpin protein; ectodomain or ectodomainΔCTT) was incubated with 50 μl of liposome (2 mg/ml) for 1 hour at 37°C. The proteins incubated with buffer only [20 mM Hepes (pH 7.5) and 150 mM NaCl] served as negative controls. The protein buffer/liposome mixtures were then centrifuged at 67,000 rpm in an ultracentrifuge (Optima TL with TLA100.4 rotor) for 20 min at 10°C. The supernatant was collected to examine proteins unbound to the liposome. The pellets were resuspended in solubilization buffer with the same volume as the supernatant. Equal amounts of supernatant and pellet were loaded for SDS–polyacrylamide gel electrophoresis analysis. Each liposome sedimentation assay was performed at least three times.

Sulforhodamine B liposome leakage assays were conducted as described (*54*). Briefly, the dried lipid mixtures as described above were rehydrated with 0.5 ml of different solubilization buffers [20 mM Hepes (pH 7.5)/50 mM citric (pH 5.5)/50 mM citric (pH 4.0)/50 mM citric (pH 3.0) supplemented with 150 mM NaCl and 50 mM sulforhodamine B]. After extrusion, the sulforhodamine B dye outside the liposome was removed by buffer exchange using a disposable PD 10 desalting column (GE Healthcare). The buffer exchange was performed twice to remove the residual sulforhodamine B outside the liposome. To perform the liposome leakage assay, the dye-encapsulated liposome was first diluted 50 times. The fluorescence of sulforhodamine B was measured with excitation wavelength of 585 nm and emission wavelength of 610 nm. The emission fluorescence before adding the proteins was used as *F*_t0_, 4 μl of protein at different concentrations was then added to 50 μl of liposomes, and the emission fluorescence was continuously measured as *F*_tn_ every 30 s for 60 min. Addition of buffer to the liposome was treated as a negative control to examine the stability of the liposomes overtime, while addition of 4 μl of 2.5% SDS to completely release the sulforhodamine B from the liposomes was treated as a positive control. The final emission fluorescence from the positive control was measured as *F*_t100_. The percentage of liposome leakage is defined as follows: Leakage (%) = (*F*_tn_ – *F*_t0_)/(*F*_t100_ – *F*_t0_) × 100. Each leakage assay was performed at least three times.

### Preparation and imaging of GUVs

GUVs were prepared by the electroformation method (*55*), as described (*56*). Lipid mixtures [POPC:POPS:*E. coli* total lipid extract (Avanti Polar Lipids) = 80:10:10 (w/w)] were dissolved in chloroform to a final concentration of 5 mg/ml. Rhodamine-DHPE (Lissamine rhodamine B 1,2-dihexadecanoyl-*sn*-glycero-3-phosphoethanolamine, triethylammonium salt) was added to fluorescently label the membranes. Six microliters of the lipid mixture was spread on the conductive side of an indium tin oxide (ITO) slide and dried under vacuum. Electroformation was carried out in sucrose solution [300 mM sucrose and 1 mM MES (pH 5.7)] between two conductive ITO slides (Vesicle Prep Pro, Nanion Technologies, Germany) under the following conditions: initial increase of amplitude from 0 to 3 V with 5-Hz frequency for 30 min, followed by 3 hours of constant amplitude of 3 V and frequency of 5 Hz, and ending with decreasing frequency from 5 to 0 Hz at a constant amplitude of 3 V at 37°C. GUVs were sedimented by first adding glucose solution [300 mM glucose and 1 mM MES (pH 5.7)], next buffer on top [10 mM MES and 150 mM NaCl (pH 5.7)], and GUVs were left to sediment overnight. All solutions used for GUV preparation and experiments were isoosmolar (Osmomat 3000, Gonotec GmbH, Germany).

GUVs were mixed with FDs of 4, 10, or 70 kDa (final concentration of 1 mg/ml) and PFN2 (final concentration of 0.1 mg/ml) and incubated for 60 or 120 min at room temperature. The buffer was used as a negative control instead of proteins. Images were recorded on a DMI6000 CS inverted microscope with TCS SP5 laser scanning system (both Leica Microsystems, Germany) with a 63× oil immersion objective (numerical aperture, 1.25). FDs and rhodamine were excited with 488- and 543-nm lasers, and fluorescence emission was detected from 495 to 530 nm and from 580 to 630 nm, respectively. Permeabilization of GUVs was quantified in ImageJ software. For each experiment, between 50 and 300 vesicles were quantified in three independent experiments.

### Planar lipid bilayer experiments

Planar lipid bilayers were prepared from a mixture POPC:POPS:*E. coli* lipid extract = 70:10:20 (w/w) with a final concentration of lipids (5 mg/ml) dissolved in octane. For recording of electrical measurements, multielectrode cavity array chips (Ionera Technologies) were used on the integrated chip-based recording setup Orbit mini with EDR2 software (Nanion Technologies). Proteins were added to the cis side of the bilayer to a final concentration of ~32 ng/μl. MES (10 mM) and NaCl (500 mM) at pH 5.5 was used as a buffer. To promote pore insertion, a voltage from ±50 mV to 200 mV with a 20-kHz sampling rate was applied. For the analysis of current, the tracing software Clampfit (10.7.0.3.) was used. The noise was quantified as described previously (*4*). In brief, the SD was determined on a short time interval (a few tens of milliseconds) of the individual current trace at which the pore was open, and noise was normalized by dividing SD with the average current of each trace at which the pore was open.

### Formation of supported lipid bilayer for HS-AFM

Vesicles were prepared using 100% *E. coli* total phospholipid extract composed of PE:PG:CA:unknown = 57.5:15.1:9.8:17.6 (w/w; Avanti, USA). Lipids dissolved in chloroform were dried under argon flux, followed by >2-hour incubation in a vacuum desiccator. After that, lipids were fully rehydrated with buffer [10 mM Hepes-NaOH (pH 7.5) and 200 mM NaCl] for 5 min at room temperature, obtaining lipid solution (0.5 mg/ml). Last, the lipid suspension was bath-sonicated for 10 min to form vesicles. For mica-supported lipid bilayers, 3 μl of *E. coli* lipid solution (0.5 mg/ml) was deposited onto freshly cleaved mica (1.5-mm-diameter disc), incubated for 15 min, and rinsed thoroughly with buffer [10 mM Hepes-NaOH (pH 7.5), 200 mM NaCl, and 2 mM CaCl_2_].

### High-speed AFM

The PFN2 assemblies on mica or on lipid bilayers were prepared by incubating PFN2 monomers (0.043 mg/ml) for 5 min, either directly on mica or on preformed supported lipid bilayers. Then, the samples were rinsed and directly imaged by HS-AFM. All images in this study were acquired using HS-AFM (SS-NEX, RIBM, Japan) operated in amplitude modulation mode using optimized scan and feedback parameters. Short (8 μm) cantilevers (NanoWorld, Switzerland) with nominal spring constant of 0.15 N/m, resonance frequency of ~0.6 MHz, and a quality factor of ∼1.5 in buffer [20 mM Hepes-NaOH (pH 7.5), 200 mM NaCl, and 2 mM CaCl_2_] were used. To study the reversible lateral displacement of pre-pores on the membrane, the ratio of set point and free amplitude (*A*_set_/*A*_free_) was changed from ~0.95 to ~0.6 and back to ~0.95. Under such conditions, the average estimated applied force on the HS-AFM tip changes from ~30 to ~200 pN and back to ~30 pN *(**57**)*. To study the pre-pore–to–pore transition, the fluid cell containing ~120 μl of buffer [20 mM Hepes-NaOH (pH 7.5), 200 mM NaCl, and 2 mM CaCl_2_] was supplemented with ~15 μl of 0.1 M HCl (through passive diffusion) to decrease the pH of the buffer solution to pH 3.6 to 4.0. The change in pH was estimated by large-scale mixing 150 μl of 0.1 M HCl into 1.20 ml of the imaging buffer.

## Supplementary Material

http://advances.sciencemag.org/cgi/content/full/6/5/eaax8286/DC1

Download PDF

Movie S1

Movie S2

Movie S3

Movie S4

Structure and mechanism of bactericidal mammalian perforin-2, an ancient agent of innate immunity

## SUPPLEMENTARY MATERIALS

Supplementary material for this article is available at http://advances.sciencemag.org/cgi/content/full/6/5/eaax8286/DC1 Fig. S1. Structure determination of mPFN2 pre-pore. Fig. S2. Structure-based phylogeny of known structures of MACPF domains from MACPF and CDC proteins. Fig. S3. Characterization of the mPFN2 P2 domain. Fig. S4. Sequence alignment of PFN2 across species. Fig. S5. pH- and concentration-dependent pore-forming activity of mPFN2. Fig. S6. pH-dependent pore-forming activity of mPFN2. Fig. S7. Disulfide locked mPFN2 pre-pores at pH 5.5. Fig. S8. Structure determination of mPFN2 pore. Table S1. Cryo-EM data collection, refinement, and validation statistics. Table S2. X-ray crystallographic statistics of P2 domain. Movie S1. Pre-pore PFN2 oligomers on mica. Movie S2. Pre-pore PFN2 oligomers on a supported lipid bilayer. Movie S3. Mobility of membrane-bound pre-pore PFN2 oligomers. Movie S4. Real-time pore formation imaged by HS-AFM.

## References

[1] Metkar S. S., Marchioretto M., Antonini V., Lunelli L., Wang B., Gilbert R. J. C., Anderluh G., Roth R., Pooga M., Pardo J., Heuser J. E., Serra M. D., Froelich C. J., Perforin oligomers form arcs in cellular membranes: A locus for intracellular delivery of granzymes. Cell Death Differ. 22, 78–85 (2015). 10.1038/cdd.2014.110 PMC4262768 25146929

[2] Leung C., Hodel A. W., Brennan A. J., Lukoyanova N., Tran S., House C. M., Kondos S. C., Whisstock J. C., Dunstone M. A., Trapani J. A., Voskoboinik I., Saibil H. R., Hoogenboom B. W., Real-time visualization of perforin nanopore assembly. Nat. Nanotechnol. 12, 467–473 (2017). 28166206 10.1038/nnano.2016.303

[3] Serna M., Giles J. L., Morgan B. P., Bubeck D., Structural basis of complement membrane attack complex formation. Nat. Commun. 7, 10587 (2016). 26841837 10.1038/ncomms10587 PMC4743022

[4] Praper T., Sonnen A., Viero G., Kladnik A., Froelich C. J., Anderluh G., Dalla Serra M., Gilbert R. J. C., Human perforin employs different avenues to damage membranes. J. Biol. Chem. 286, 2946–2955 (2011). 20889983 10.1074/jbc.M110.169417 PMC3024789

[5] Gilbert R. J. C., Dalla Serra M., Froelich C. J., Wallace M. I., Anderluh G., Membrane pore formation at protein–lipid interfaces. Trends Biochem. Sci. 39, 510–516 (2014). 25440714 10.1016/j.tibs.2014.09.002

[6] Wiens M., Korzhev M., Krasko A., Thakur N. L., Perović-Ottstadt S., Breter H. J., Ushijima H., Diehl-Seifert B., Müller I. M., Müller W. E. G., Innate immune defense of the sponge *Suberites domuncula* against bacteria involves a MyD88-dependent signaling pathway. Induction of a perforin-like molecule. J. Biol. Chem. 280, 27949–27959 (2005). 15923643 10.1074/jbc.M504049200

[7] McCormack R., Podack E. R., Perforin-2/Mpeg1 and other pore-forming proteins throughout evolution. J. Leukoc. Biol. 98, 761–768 (2015). 26307549 10.1189/jlb.4MR1114-523RR PMC4600061

[8] Benard E. L., Racz P. I., Rougeot J., Nezhinsky A. E., Verbeek F. J., Spaink H. P., Meijer A. H., Macrophage-expressed perforins mpeg1 and mpeg1.2 have an anti-bacterial function in zebrafish. J. Innate Immun. 7, 136–152 (2015). 25247677 10.1159/000366103 PMC6738794

[9] D’Angelo M. E., Dunstone M. A., Whisstock J. C., Trapani J. A., Bird P. I., Perforin evolved from a gene duplication of MPEG1, followed by a complex pattern of gene gain and loss within *Euteleostomi*. BMC Evol. Biol. 12, 59 (2012). 22551122 10.1186/1471-2148-12-59 PMC3477005

[10] McCormack R., de Armas L. R., Shiratsuchi M., Ramos J. E., Podack E. R., Inhibition of intracellular bacterial replication in fibroblasts is dependent on the perforin-like protein (perforin-2) encoded by macrophage-expressed gene 1. J. Innate Immun. 5, 185–194 (2013). 23257510 10.1159/000345249 PMC3732477

[11] Fields K. A., McCormack R., de Armas L. R., Podack E. R., Perforin-2 restricts growth of *Chlamydia trachomatis* in macrophages. Infect. Immun. 81, 3045–3054 (2013). 23753625 10.1128/IAI.00497-13 PMC3719555

[12] McCormack R. M., de Armas L. R., Shiratsuchi M., Fiorentino D. G., Olsson M. L., Lichtenheld M. G., Morales A., Lyapichev K., Gonzalez L. E., Strbo N., Sukumar N., Stojadinovic O., Plano G. V., Munson G. P., Tomic-Canic M., Kirsner R. S., Russell D. G., Podack E. R., Perforin-2 is essential for intracellular defense of parenchymal cells and phagocytes against pathogenic bacteria. Elife 4, e06508 (2015). 26402460 10.7554/eLife.06508 PMC4626811

[13] McCormack R. M., Lyapichev K., Olsson M. L., Podack E. R., Munson G. P., Enteric pathogens deploy cell cycle inhibiting factors to block the bactericidal activity of Perforin-2. Elife 4, e06505 (2015). 26418746 10.7554/eLife.06505 PMC4626573

[14] Botto M., Kirschfink M., Macor P., Pickering M. C., Würzner R., Tedesco F., Complement in human diseases: Lessons from complement deficiencies. Mol. Immunol. 46, 2774–2783 (2009). 19481265 10.1016/j.molimm.2009.04.029

[15] McCormack R. M., Szymanski E. P., Hsu A. P., Perez E., Olivier K. N., Fisher E., Goodhew E. B., Podack E. R., Holland S. M., *MPEG1*/perforin-2 mutations in human pulmonary nontuberculous mycobacterial infections. JCI Insight 2, e89635 (2017). 10.1172/jci.insight.89635 PMC5396519 28422754

[16] McCormack R., Bahnan W., Shrestha N., Boucher J., Barreto M., Barrera C. M., Dauer E. A., Freitag N. E., Khan W. N., Podack E. R., Schesser K., Perforin-2 protects host cells and mice by restricting the vacuole to cytosol transitioning of a bacterial pathogen. Infect. Immun. 84, 1083–1091 (2016). 26831467 10.1128/IAI.01434-15 PMC4807494

[17] Ni T., Williams S. I., Rezelj S., Anderluh G., Harlos K., Stansfeld P. J., Gilbert R. J. C., Structures of monomeric and oligomeric forms of the *Toxoplasma gondii* perforin-like protein 1. Sci. Adv. 4, eaaq0762 (2018). 29750191 10.1126/sciadv.aaq0762 PMC5943054

[18] Tilley S. J., Orlova E. V., Gilbert R. J., Andrew P. W., Saibil H. R., Structural basis of pore formation by the bacterial toxin pneumolysin. Cell 121, 247–256 (2005). 15851031 10.1016/j.cell.2005.02.033

[19] van Pee K., Neuhaus A., D’Imprima E., Mills D. J., Kühlbrandt W., Yildiz Ö., CryoEM structures of membrane pore and prepore complex reveal cytolytic mechanism of Pneumolysin. Elife 6, e23644 (2017). 28323617 10.7554/eLife.23644 PMC5437283

[20] Miyagi A., Chipot C., Rangl M., Scheuring S., High-speed atomic force microscopy shows that annexin V stabilizes membranes on the second timescale. Nat. Nanotechnol. 11, 783–790 (2016). 27271964 10.1038/nnano.2016.89

[21] Metkar S. S., Wang B., Catalan E., Anderluh G., Gilbert R. J. C., Pardo J., Froelich C. J., Perforin rapidly induces plasma membrane phospholipid flip-flop. PLOS ONE 6, e24286 (2011). 21931672 10.1371/journal.pone.0024286 PMC3171411

[22] Sonnen A. F. P., Plitzko J. M., Gilbert R. J. C., Incomplete pneumolysin oligomers form membrane pores. Open Biol. 4, 140044 (2014). 24759615 10.1098/rsob.140044 PMC4043118

[23] Lukoyanova N., Hoogenboom B. W., Saibil H. R., The membrane attack complex, perforin and cholesterol-dependent cytolysin superfamily of pore-forming proteins. J. Cell Sci. 129, 2125–2133 (2016). 27179071 10.1242/jcs.182741

[24] G. Anderluh, R. J. C. Gilbert, MACPF/CDC proteins—Agents of defence, attack and invasion, in *Subcellular Biochemistry*, J. R. Harris, Ed. (Springer, 2014), vol. 80.

[25] Podobnik M., Savory P., Rojko N., Kisovec M., Wood N., Hambley R., Pugh J., Wallace E. J., McNeill L., Bruce M., Liko I., Allison T. M., Mehmood S., Yilmaz N., Kobayashi T., Gilbert R. J. C., Robinson C. V., Jayasinghe L., Anderluh G., Crystal structure of an invertebrate cytolysin pore reveals unique properties and mechanism of assembly. Nat. Commun. 7, 11598 (2016). 27176125 10.1038/ncomms11598 PMC4865846

[26] Iacovache I., De Carlo S., Cirauqui N., Dal Peraro M., van der Goot F. G., Zuber B., Cryo-EM structure of aerolysin variants reveals a novel protein fold and the pore-formation process. Nat. Commun. 7, 12062 (2016). 27405240 10.1038/ncomms12062 PMC4947156

[27] Shatursky O., Heuck A. P., Shepard L. A., Rossjohn J., Parker M. W., Johnson A. E., Tweten R. K., The mechanism of membrane insertion for a cholesterol-dependent Cytolysin. Cell 99, 293–299 (1999). 10555145 10.1016/s0092-8674(00)81660-8

[28] Law R. H. P., Lukoyanova N., Voskoboinik I., Caradoc-Davies T. T., Baran K., Dunstone M. A., D’Angelo M. E., Orlova E. V., Coulibaly F., Verschoor S., Browne K. A., Ciccone A., Kuiper M. J., Bird P. I., Trapani J. A., Saibil H. R., Whisstock J. C., The structural basis for membrane binding and pore formation by lymphocyte perforin. Nature 468, 447–451 (2010). 21037563 10.1038/nature09518

[29] Ni T., Gilbert R. J. C., Repurposing a pore: Highly conserved perforin-like proteins with alternative mechanisms. Philos. Trans. R. Soc. Lond. B Biol. Sci. 372, 20160212 (2017). 28630152 10.1098/rstb.2016.0212 PMC5483515

[30] Ni T., Harlos K., Gilbert R., Structure of astrotactin-2: A conserved vertebrate-specific and perforin-like membrane protein involved in neuronal development. Open Biol. 6, 160053 (2016). 27249642 10.1098/rsob.160053 PMC4892435

[31] Rossjohn J., Feil S. C., McKinstry W. J., Tweten R. K., Parker M. W., Structure of a cholesterol-binding, thiol-activated cytolysin and a model of its membrane form. Cell 89, 685–692 (1997). 9182756 10.1016/s0092-8674(00)80251-2

[32] Aricescu A. R., Assenberg R., Bill R. M., Busso D., Chang V. T., Davis S. J., Dubrovsky A., Gustafsson L., Hedfalk K., Heinemann U., Jones I. M., Ksiazek D., Lang C., Maskos K., Messerschmidt A., Macieira S., Peleg Y., Perrakis A., Poterszman A., Schneider G., Sixma T. K., Sussman J. L., Sutton G., Tarboureich N., Zeev-Ben-Mordehai T., Jones E. Y., Eukaryotic expression: Developments for structural proteomics. Acta Crystallogr. D Biol. Crystallogr. 62, 1114–1124 (2006). 17001089 10.1107/S0907444906029805 PMC7161643

[33] Molday L. L., Molday R. S., 1D4: A versatile epitope tag for the purification and characterization of expressed membrane and soluble proteins. Methods Mol. Biol. 1177, 1–15 (2014). 24943310 10.1007/978-1-4939-1034-2_1 PMC4227631

[34] Zheng S. Q., Palovcak E., Armache J.-P., Verba K. A., Cheng Y., Agard D. A., MotionCor2: Anisotropic correction of beam-induced motion for improved cryo-electron microscopy. Nat. Methods 14, 331–332 (2017). 28250466 10.1038/nmeth.4193 PMC5494038

[35] Zhang K., Gctf: Real-time CTF determination and correction. J. Struct. Biol. 193, 1–12 (2016). 26592709 10.1016/j.jsb.2015.11.003 PMC4711343

[36] de la Rosa-Trevin J. M., Otón J., Marabini R., Zaldívar A., Vargas J., Carazo J. M., Sorzano C. O. S., Xmipp 3.0: An improved software suite for image processing in electron microscopy. J. Struct. Biol. 184, 321–328 (2013). 24075951 10.1016/j.jsb.2013.09.015

[37] Scheres S. H. W., RELION: Implementation of a Bayesian approach to cryo-EM structure determination. J. Struct. Biol. 180, 519–530 (2012). 23000701 10.1016/j.jsb.2012.09.006 PMC3690530

[38] Punjani A., Rubinstein J. L., Fleet D. J., Brubaker M. A., cryoSPARC: Algorithms for rapid unsupervised cryo-EM structure determination. Nat. Methods 14, 290–296 (2017). 28165473 10.1038/nmeth.4169

[39] Pettersen E. F., Goddard T. D., Huang C. C., Couch G. S., Greenblatt D. M., Meng E. C., Ferrin T. E., UCSF chimera—A visualization system for exploratory research and analysis. J. Comput. Chem. 25, 1605–1612 (2004). 15264254 10.1002/jcc.20084

[40] Kelley L. A., Mezulis S., Yates C. M., Wass M. N., Sternberg M. J. E., The Phyre2 web portal for protein modeling, prediction and analysis. Nat. Protoc. 10, 845–858 (2015). 25950237 10.1038/nprot.2015.053 PMC5298202

[41] Emsley P., Cowtan K., *Coot*: Model-building tools for molecular graphics. Acta Crystallogr. D Biol. Crystallogr. 60, 2126–2132 (2004). 15572765 10.1107/S0907444904019158

[42] Afonine P. V., Grosse-Kunstleve R. W., Echols N., Headd J. J., Moriarty N. W., Mustyakimov M., Terwilliger T. C., Urzhumtsev A., Zwart P. H., Adams P. D., Towards automated crystallographic structure refinement with *phenix.refine*. Acta Crystallogr. D Biol. Crystallogr. 68, 352–367 (2012). 22505256 10.1107/S0907444912001308 PMC3322595

[43] Lopez-Blanco J. R., Chacón P., iMODFIT: Efficient and robust flexible fitting based on vibrational analysis in internal coordinates. J. Struct. Biol. 184, 261–270 (2013). 23999189 10.1016/j.jsb.2013.08.010

[44] Kidmose R. T., Juhl J., Nissen P., Boesen T., Karlsen J. L., Pedersen B. P., Namdinator—Automatic molecular dynamics flexible fitting of structural models into cryo-EM and crystallography experimental maps. IUCrJ 6, 526–531 (2019). 10.1107/S2052252519007619 PMC6608625 31316797

[45] Trabuco L. G., Villa E., Schreiner E., Harrison C. B., Schulten K., Molecular dynamics flexible fitting: A practical guide to combine cryo-electron microscopy and x-ray crystallography. Methods 49, 174–180 (2009). 19398010 10.1016/j.ymeth.2009.04.005 PMC2753685

[46] Stuart D. I., Levine M., Muirhead H., Stammers D. K., Crystal structure of cat muscle pyruvate kinase at a resolution of 2.6 Å. J. Mol. Biol. 134, 109–142 (1979). 537059 10.1016/0022-2836(79)90416-9

[47] Riffel N., Harlos K., Iourin O., Rao Z., Kingsman A., Stuart D., Fry E., Atomic resolution structure of Moloney murine leukemia virus matrix protein and its relationship to other retroviral matrix proteins. Structure 10, 1627–1636 (2002). 12467570 10.1016/s0969-2126(02)00896-1

[48] Felsenstein J., An alternating least squares approach to inferring phylogenies from pairwise distances. Syst. Biol. 46, 101–111 (1997). 11975348 10.1093/sysbio/46.1.101

[49] Pape T., Schneider G., *HKL2MAP*: A graphical user interface for macromolecular phasing with *SHELX* programs. J. Appl. Cryst. 37, 843–844 (2004).

[50] Terwilliger T. C., Adams P. D., Read R. J., McCoy A., Moriarty N. W., Grosse-Kunstleve R. W., Afonine P. V., Zwart P. H., Hung L.-W., Decision-making in structure solution using Bayesian estimates of map quality: The *PHENIX AutoSol* wizard. Acta Crystallogr. D Biol. Crystallogr. 65, 582–601 (2009). 19465773 10.1107/S0907444909012098 PMC2685735

[51] Terwilliger T. C., Grosse-Kunstleve R. W., Afonine P. V., Moriarty N. W., Zwart P. H., Hung L.-W., Read R. J., Adams P. D., Iterative model building, structure refinement and density modification with the *PHENIX AutoBuild* wizard. Acta Crystallogr. D Biol. Crystallogr. 64, 61–69 (2008). 18094468 10.1107/S090744490705024X PMC2394820

[52] Tang G., Peng L., Baldwin P. R., Mann D. S., Jiang W., Rees I., Ludtke S. J., EMAN2: An extensible image processing suite for electron microscopy. J. Struct. Biol. 157, 38–46 (2007). 16859925 10.1016/j.jsb.2006.05.009

[53] van Heel M., Harauz G., Orlova E. V., Schmidt R., Schatz M., A new generation of the IMAGIC image processing system. J. Struct. Biol. 116, 17–24 (1996). 8742718 10.1006/jsbi.1996.0004

[54] Faudry E., Perdu C., Attrée I., Pore formation by T3SS translocators: Liposome leakage assay. Methods Mol. Biol. 966, 173–185 (2013). 23299735 10.1007/978-1-62703-245-2_11

[55] Angelova M. I., Dimitov D. S., Liposome electroformation. Faraday Discuss. 81, 303–311 (1986).

[56] Ruan Y., Rezelj S., Bedina Zavec A., Anderluh G., Scheuring S., Listeriolysin O membrane damaging activity involves arc formation and lineaction—Implication for *Listeria monocytogenes* escape from phagocytic vacuole. PLOS Pathog. 12, e1005597 (2016). 27104344 10.1371/journal.ppat.1005597 PMC4841516

[57] Miyagi A., Scheuring S., Automated force controller for amplitude modulation atomic force microscopy. Rev. Sci. Instrum. 87, 053705 (2016). 27250433 10.1063/1.4950777

