# Supplementary material for: Structure and mechanism of bactericidal mammalian perforin-2, an ancient agent of innate immunity
Source: Sci Adv. 2020 Jan 29;6(5):eaax8286. doi: 10.1126/sciadv.aax8286 (PMC6989145; doi:10.1126/sciadv.aax8286)
Supplement: http://advances.sciencemag.org/cgi/content/full/6/5/eaax8286/DC1 [file supp_6_5_eaax8286__index.html]

Science Advances | Science AdvancesAAASSearchScience AdvancesMenu

## Supplementary Materials

**The PDFset includes:**

- Fig. S1. Structure determination of mPFN2 pre-pore.
- Fig. S2. Structure-based phylogeny of known structures of MACPF domains from MACPF and CDC proteins.
- Fig. S3. Characterization of the mPFN2 P2 domain.
- Fig. S4. Sequence alignment of PFN2 across species.
- Fig. S5. pH- and concentration-dependent pore-forming activity of mPFN2.
- Fig. S6. pH-dependent pore-forming activity of mPFN2.
- Fig. S7. Disulfide locked mPFN2 pre-pores at pH 5.5.
- Fig. S8. Structure determination of mPFN2 pore.
- Table S1. Cryo-EM data collection, refinement, and validation statistics.
- Table S2. X-ray crystallographic statistics of P2 domain.
- Legends for movies S1 to S4

Download PDF

**Other Supplementary Material for this manuscript includes the following:**

- Movie S1 (.mov format). Pre-pore PFN2 oligomers on mica.
- Movie S2 (.mov format). Pre-pore PFN2 oligomers on a supported lipid bilayer.
- Movie S3 (.mov format). Mobility of membrane-bound pre-pore PFN2 oligomers.
- Movie S4 (.mov format). Real-time pore formation imaged by HS-AFM.

**Files in this Data Supplement:**

- Adobe PDF - aax8286\_SM.pdf
