# Supplementary material for: Structure and mechanism of bactericidal mammalian perforin-2, an ancient agent of innate immunity
Source: Sci Adv. 2020 Jan 29;6(5):eaax8286. doi: 10.1126/sciadv.aax8286 (PMC6989145; doi:10.1126/sciadv.aax8286)
Supplement: Download PDF [file aax8286_SM.pdf]

Science Advances
AAAS

advances.sciencemag.org/cgi/content/full/6/5/eaax8286/DC1

Supplementary Materials for

Structure and mechanism of bactericidal mammalian perforin-2, an ancient agent of innate immunity

Tao Ni, Fang Jiao, Xiulian Yu, Saša Aden, Lucy Ginger, Sophie I. Williams, Fangfang Bai, Vojtěch Pražák, Dimple Karia, Phillip Stansfeld, Peijun Zhang, George Munson, Gregor Anderluh, Simon Scheuring*, Robert J. C. Gilbert*

*Corresponding author. Email: sis2019@med.cornell.edu (S.S.); gilbert@strubi.ox.ac.uk (R.J.C.G.)

Published 29 January 2020, *Sci. Adv.* **6**, eaax8286 (2020)
DOI: 10.1126/sciadv.aax8286

The PDF file includes:

Fig. S1. Structure determination of mPFN2 pre-pore.
Fig. S2. Structure-based phylogeny of known structures of MACPF domains from MACPF and CDC proteins.
Fig. S3. Characterization of the mPFN2 P2 domain.
Fig. S4. Sequence alignment of PFN2 across species.
Fig. S5. pH- and concentration-dependent pore-forming activity of mPFN2.
Fig. S6. pH-dependent pore-forming activity of mPFN2.
Fig. S7. Disulfide locked mPFN2 pre-pores at pH 5.5.
Fig. S8. Structure determination of mPFN2 pore.
Table S1. Cryo-EM data collection, refinement, and validation statistics.
Table S2. X-ray crystallographic statistics of P2 domain.
Legends for movies S1 to S4

Other Supplementary Material for this manuscript includes the following:

(available at advances.sciencemag.org/cgi/content/full/6/5/eaax8286/DC1)

Movie S1 (.mov format). Pre-pore PFN2 oligomers on mica.
Movie S2 (.mov format). Pre-pore PFN2 oligomers on a supported lipid bilayer.
Movie S3 (.mov format). Mobility of membrane-bound pre-pore PFN2 oligomers.
Movie S4 (.mov format). Real-time pore formation imaged by HS-AFM.

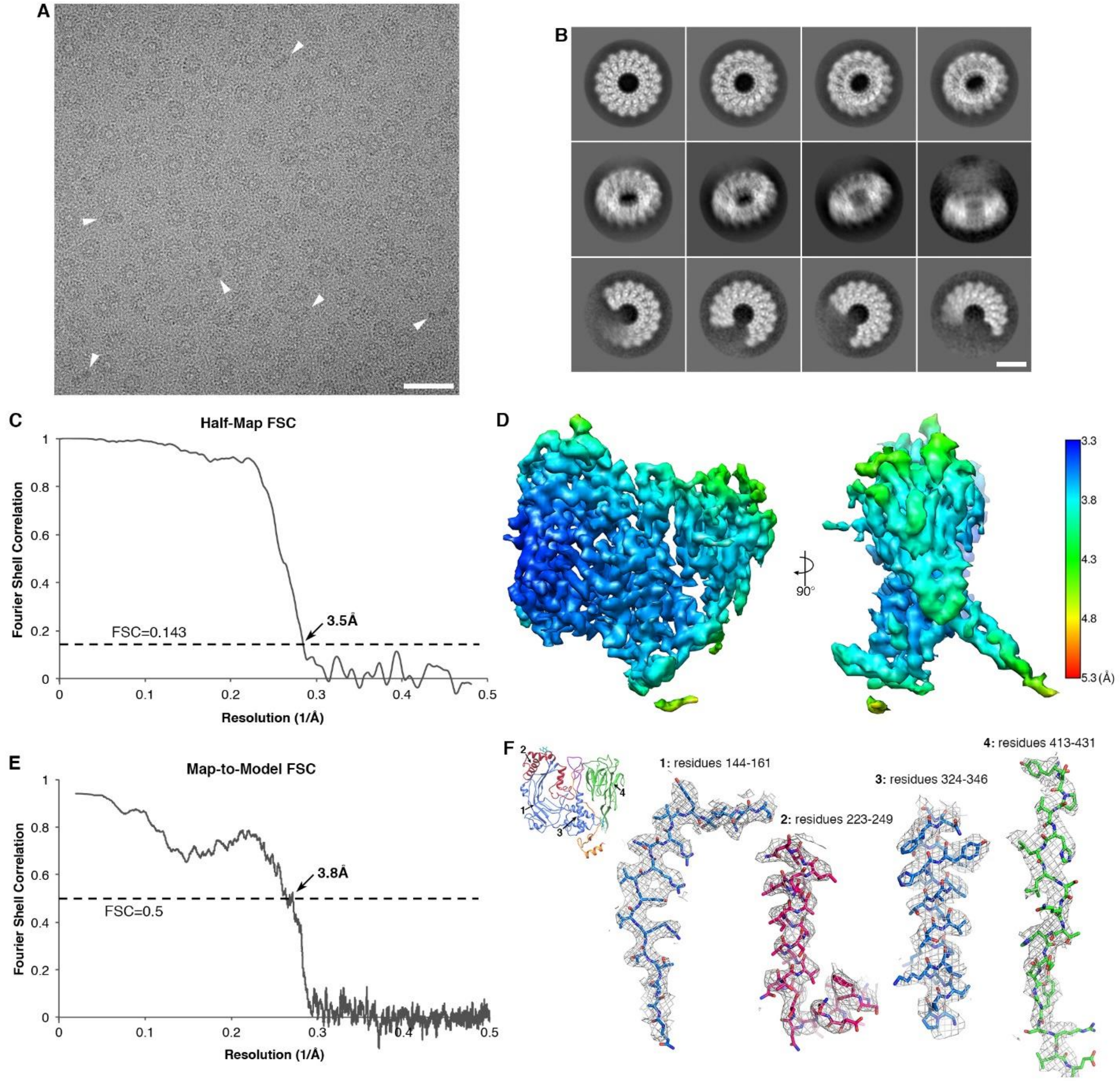

Fig. S1. Structure determination of mPFN2 pre-pore. (**A**) Representative cryo-EM micrograph of mPFN2 pre-pore oligomer, arrowheads indicate the pre-pore side views. Bar = 50 nm. (**B**) Two-dimensional class averages of the mPFN2 pre-pore in multiple orientations indicating also the presence of arcs of subunits as well as full rings. Bar = 10 nm. (**C**) Global resolution assessment of the pre-pore map measured using the 'Gold-standard' Fourier shell correlation (FSC) curves from two half-reconstructions refined separately in RELION. (**D**) Local resolution evaluation of the mPFN2 protomer by RELION. (**E**) Map-to-Model FSC curves showing the correlations between the final atomic model and the final cryo-EM map. (**F**) Representative regions of cryo-EM densities (mesh) superposed with our atomic model and colored as in Fig. 1.

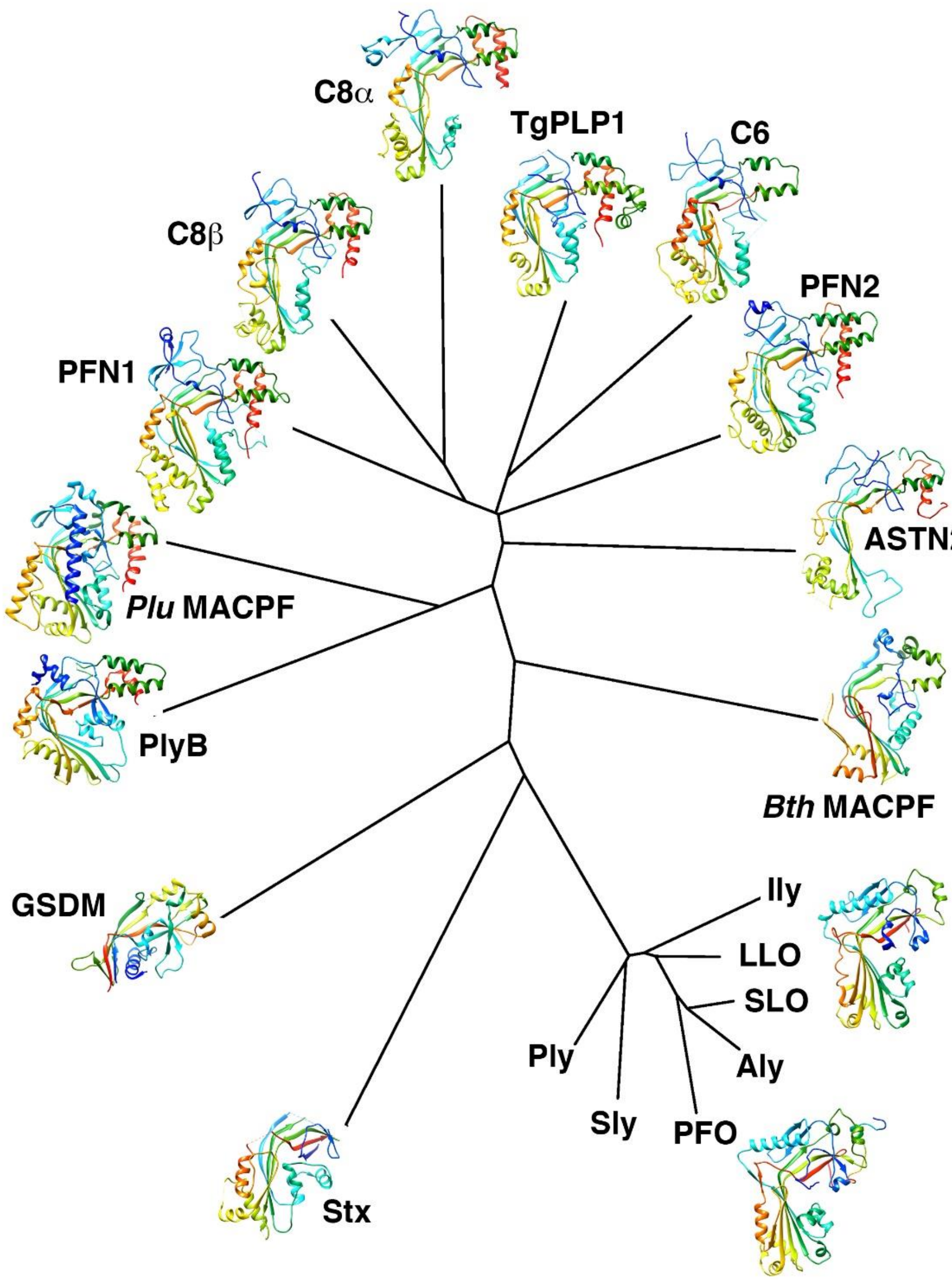

Fig. S2. Structure-based phylogeny of known structures of MACPF domains from MACPF and CDC proteins. In addition to PFN2 we show astrotactin-2 (ASTN2), the *Bacillus thetaiotaomicron* MACPF domain (*Bth*), stonefish stonustoxin (Stx), gasdermin A (GSDM), pleurotolysin B (PlyB), *Photorhabdus luminescens* MACPF (*Plu*), perforin-1 (PFN1), complement proteins C6, C8α and C8β, *Toxoplasma gondii* perforin-like protein-1 (TgPLP1) and the CDCs pneumolysin (Ply), suilysin (Sly), perfringolysin O (PFO; structure shown), anthrolysin (Aly), streptolysin O (SLO), listeriolysin (LLO; structure shown) and intermedilysin (Ily).

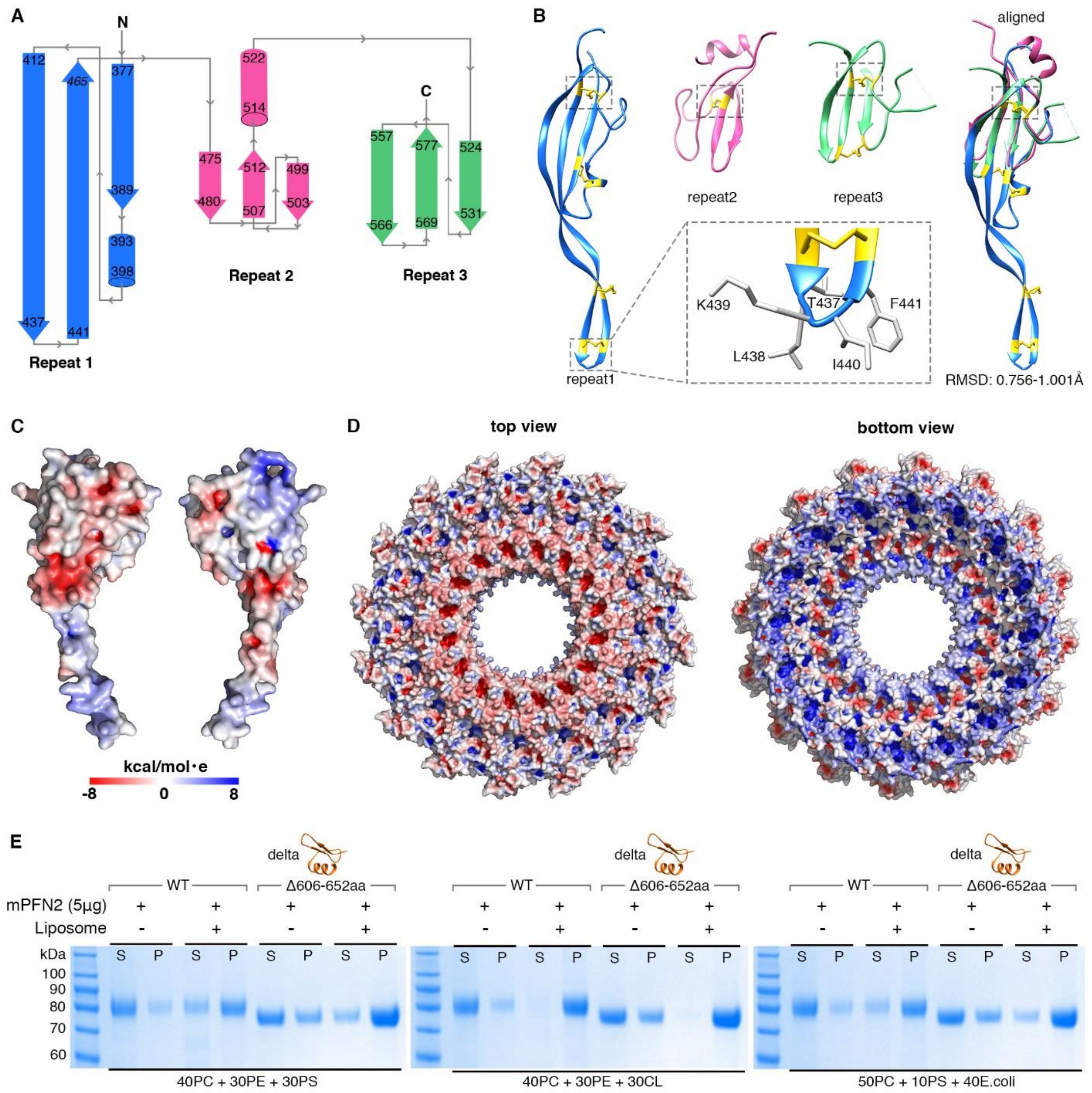

Fig. S3. Characterization of the mPFN2 P2 domain. (**A**) Structure topology of mPFN2 P2 domain showing three repeated modules. (**B**) Superposition of the three tandem repeats showing the conservation of the three strands and a disulfide bond from each repeat (RMSD= 0.756-1.001 Å). The three repeats were colored in blue, pink and green respectively with the conserved disulfide bond (in yellow) indicated by the dashed boxes. The enlarged box represents the residues (TLKIF) forming the tip of the first repeat. (**C**) electrostatic surface potential for the P2 domain crystal structure and (**D**) for the pre-pore showing marked concentrations of positive and negative potential, with the positively-charged regions in places which will interact with negatively-charged lipid headgroups on membrane binding. (**E**) Ultracentrifugation-based liposome-binding assays of mPFN2 ectodomain and ectodomain mutant with CTTΔ606-652aa truncation.

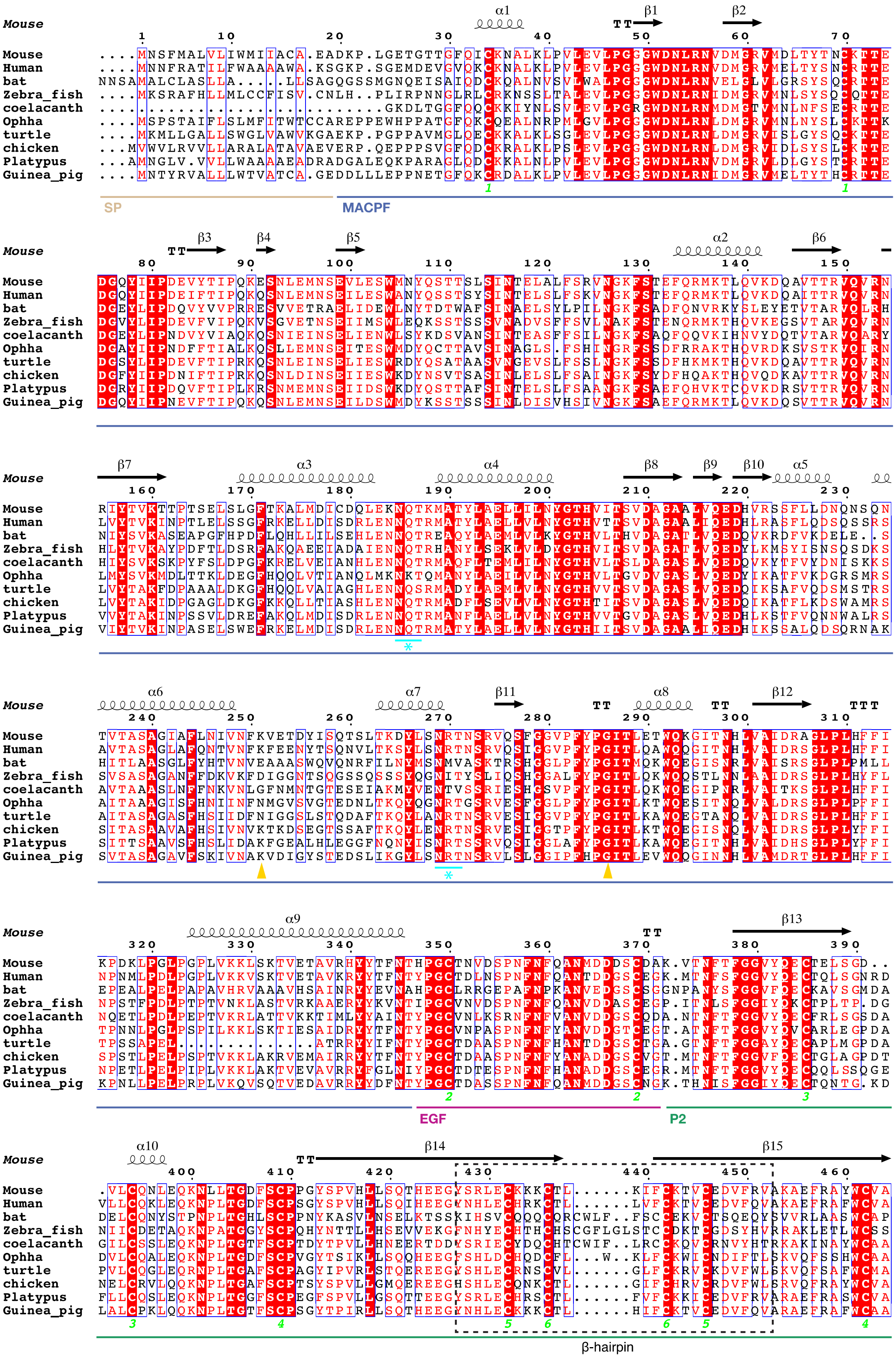

Mouse
Human
bat
Zebra_fish
coelacanth
Ophha
turtle
chicken
Platypus
Guinea_pig
SP
MACPF
EGF
P2
β-hairpin

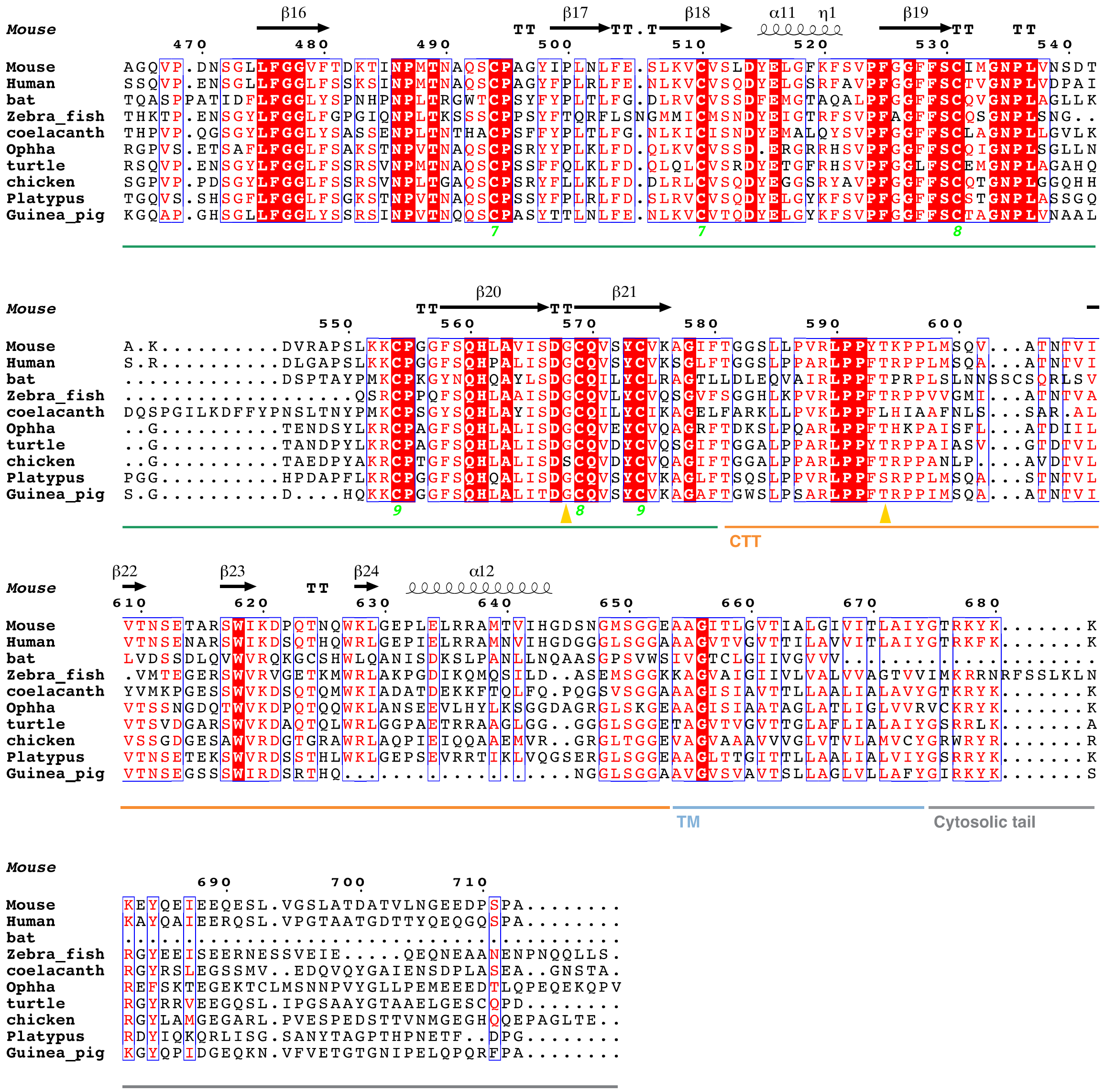

Fig. S4. Sequence alignment of PFN2 across species. Sequence alignment of PFN2 based on amino acid sequences taken from Uniprot: mouse (A1L314), human (Q2M385), bat (G1QCG4), zebrafish (Q7SXE0), coelacanth (H3A4Y7), ophha (V8NKD5), turtle (K7FJT2), chicken (R4GL52), platypus (F6W8W1) and guinea pig (H0VL25). Numbers above the sequence alignment correspond to amino acid residue numbers relative to the mouse PFN2 sequence, where residue 1 is the initial methionine. The pre-pore secondary structures determined from mouse PFN2 are marked along the sequence. Different domain sequences are indicated by lines color-coded as in Fig. 1. The dashed box between β14 and β15 represents the β-hairpin of the P2 domain. The mutated residues forming the disulfide lock are indicated by gold arrowheads. The conserved N-glycosylation sites are labeled with cyan asterisks below cyan lines.

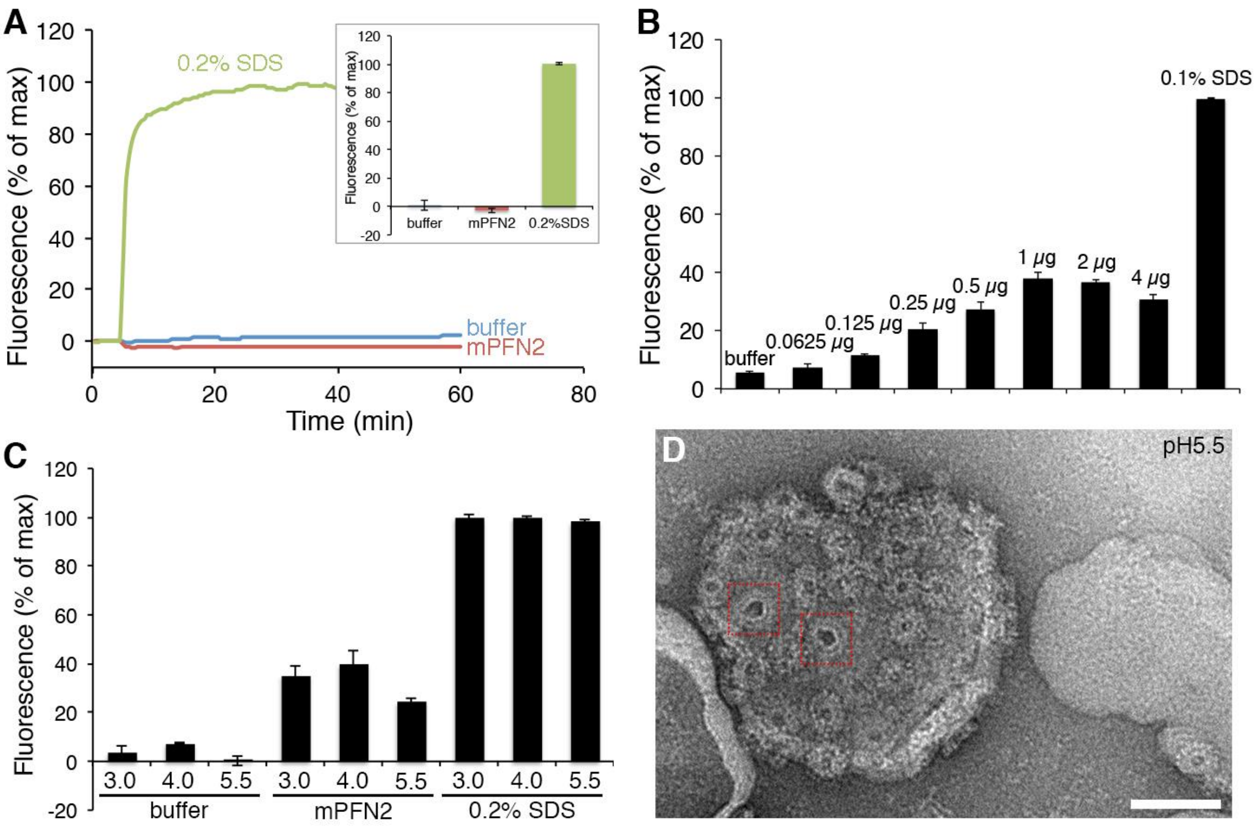

Fig. S5. pH- and concentration-dependent pore-forming activity of mPFN2. (**A**) Liposome leakage assay of purified mPFN2 protein incubated with liposomes containing 50%PC, 10%PS and 40% *E.coli* total extract at pH7.5 showed no pore forming activity. The insert shows final leakage of sulforhodamine B as mean ± s.d. from three technical replicates. (**B**) Final leakage of sulforhodamine B from liposomes incubated with different concentrations of mPFN2 as shown in Fig. 4A, expressed as mean ± s.d. from three technical replicates. (**C**) Final leakage of sulforhodamine B from liposomes pre-formed at different pH values as shown in Fig. 4B, expressed as mean ± s.d. from three technical replicates. (**D**) Negative stain micrograph showing a few mPFN2 pores (boxed) formed on liposomes at pH5.5. Bar = 50nm.

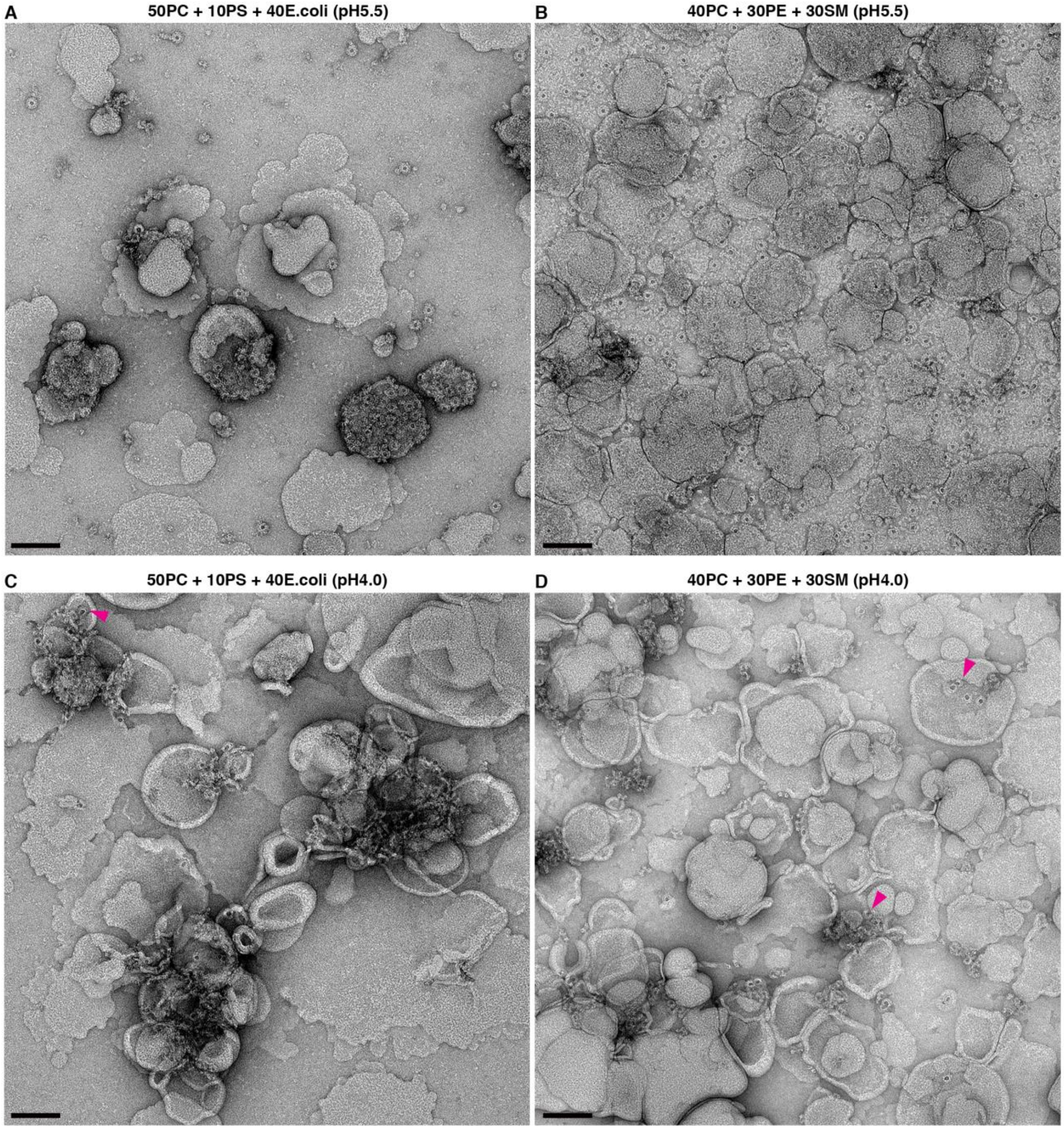

Fig. S6. pH-dependent pore-forming activity of mPFN2. (**A** and **B**) Representative negative-stain micrographs showing that at pH5.5, the amount of mPFN2 pre-pore binding to a liposome containing 30%SM is dramatically reduced (B), compared to that bound to the liposome containing 40% *E.coli* total lipid extract (A). (**C**) Representative negative-stain micrograph of mPFN2 incubated with liposomes containing 40% *E.coli* total lipid extract at pH4.0, showing the clustering of the liposomes. (**D**) Representative negative-stain micrograph of mPFN2 incubated with liposomes containing 30% SM at pH4.0, showing more distributed pore oligomers without clustering of the liposomes. Magenta arrowheads show the presence of arcs in (C) and (D). Bar = 100 nm.

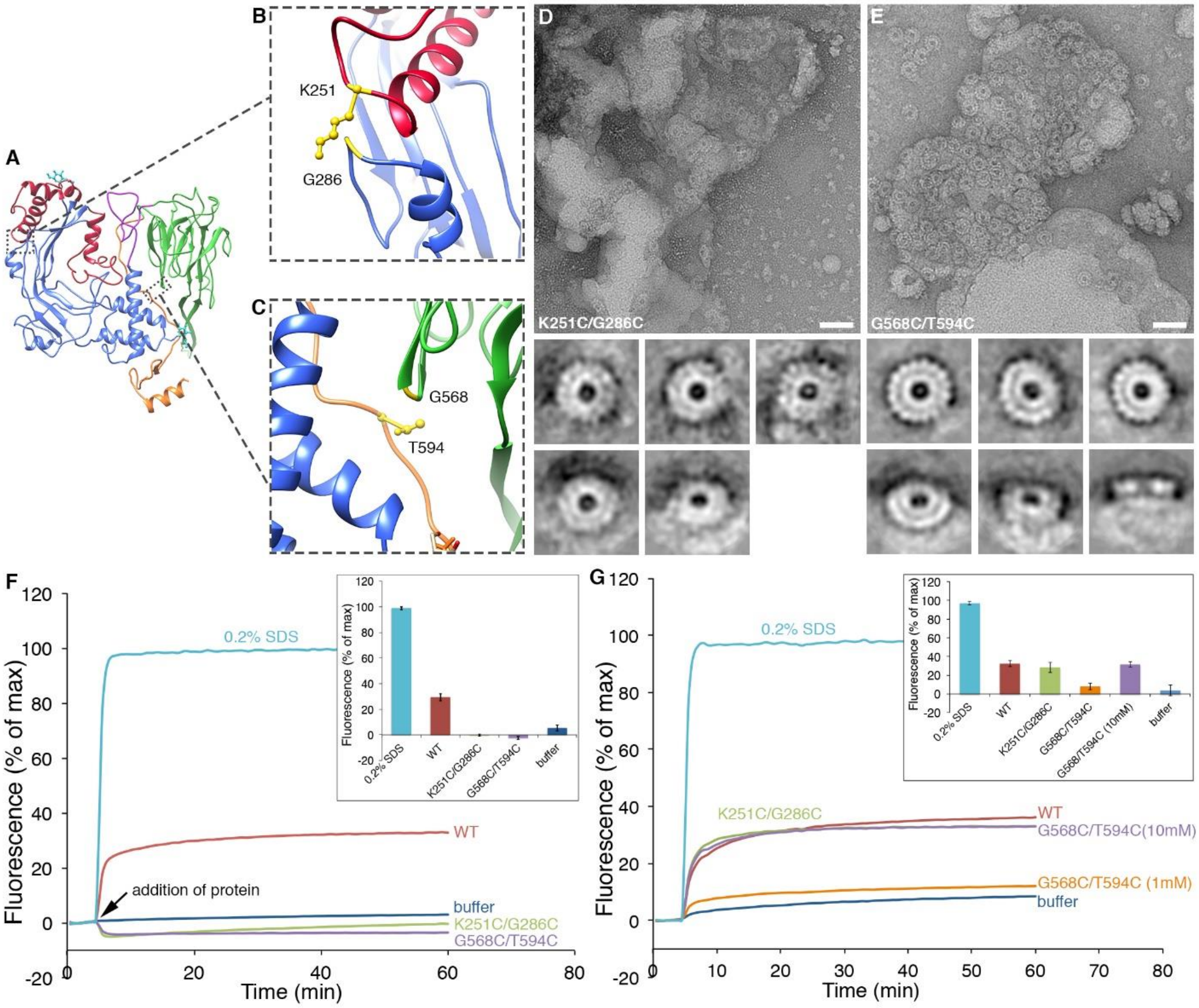

Fig. S7. Disulfide locked mPFN2 pre-pores at pH 5.5. (**A-C**) Close-up views of the TMH2 helix disulfide lock (B) and P2-CTT inter-domain lock (C) as boxed in (A). (**D**, **E**) Representative negative-stain micrograph and 2D class averages of oxidized PFN2$^{K251C/G286C}$ and PFN2$^{G568C/T594C}$ incubated with liposomes at pH5.5. (**F**) Sulforhodamine B release from liposomes incubated with mPFN2^{WT}, PFN2$^{K251C/G286C}$ and PFN2$^{G568C/T594C}$ respectively indicating the blockage of pore formation by the introduced mutation at pH5.5. The insert shows final leakage of sulforhodamine B expressed as mean ± s.d. from three technical replicates. (**G**) Pre-incubation of PFN2$^{K251C/G286C}$ mutant with 1mM DTT restored the pore forming activity of mPFN2, while 10mM DTT was required for restoring the pore forming activity of the PFN2$^{G568C/T594C}$ mutant. Final leakage of sulforhodamine B represented as mean ± s.d. from three technical replicates is shown as an insert. All data shown are representative of three independent experiments.

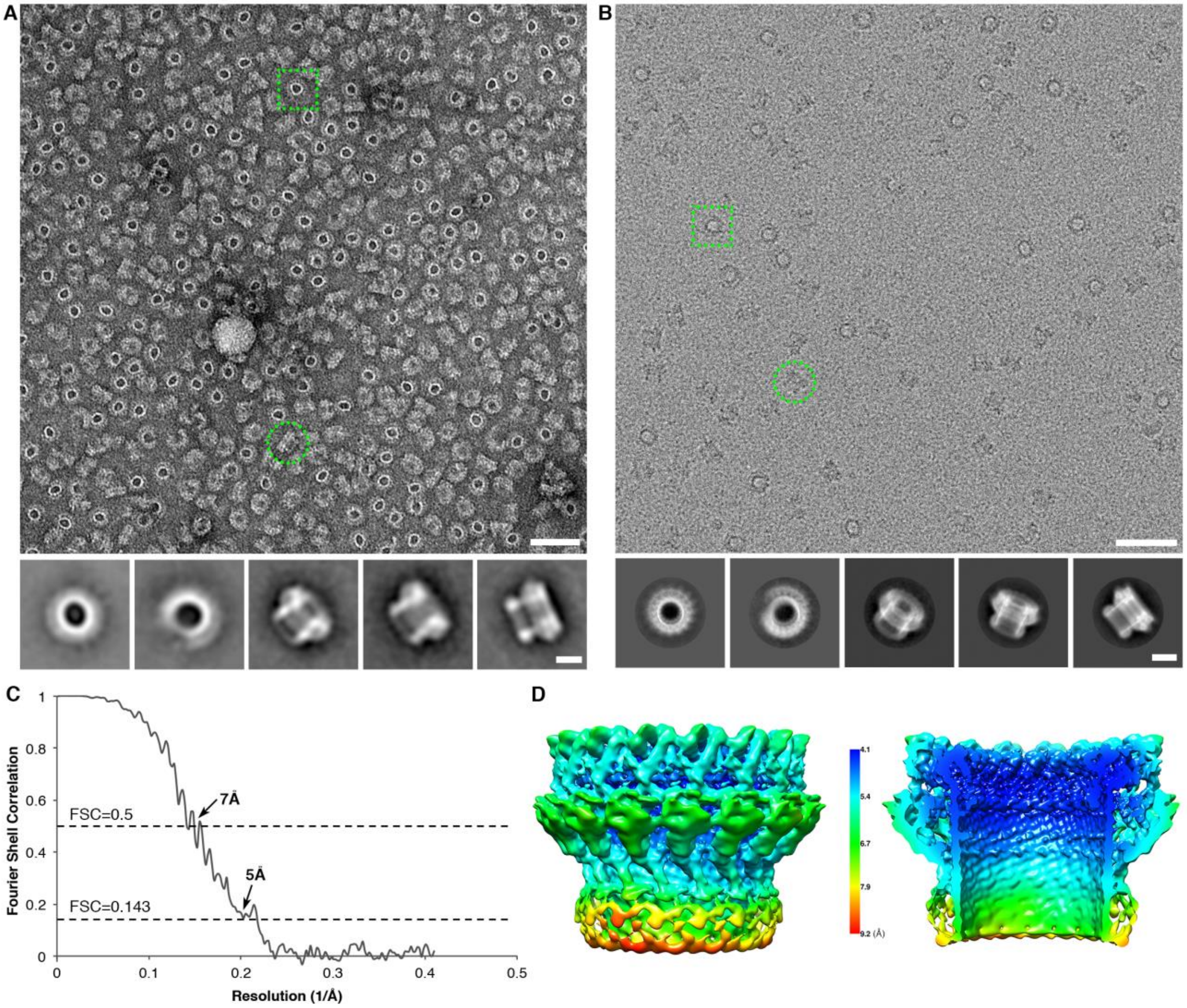

Fig. S8. Structure determination of mPFN2 pore. (**A**) Representative negative-stain micrograph and 2D class averages of mPFN2 pore. Representative top view and side view particles are indicated with a green square and a circle, respectively. (**B**) Representative cryo-EM micrograph and 2D class averages of mPFN2 pore oligomers, a green square and circle indicate the top and side views of the pore, respectively. Bars represent 50 nm in the micrograph and 10 nm in the 2D class averages in A and B. (**C**) Global resolution assessment of the pore map measured using the 'Gold-standard' Fourier shell correlation (FSC) curves from two half-reconstructions refined separately in RELION. (**D**) Local resolution evaluation of the mPFN2 pore by RELION.

Table S1. Cryo-EM data collection, refinement, and validation statistics.

	Pre-pore dataset (EMDB-10134) (PDB 6SB3)	Pore dataset (EMDB-10135) (PDB 6SB5)
**Data collection and processing**		
Magnification	48077	40983
Voltage (kV)	300	200
Electron exposure (e–/Å^2)	40	45
Defocus range (μm)	-1 - -2.5	-1 - -2.5
Pixel size (Å)	1.04	1.22
Symmetry imposed	C16	C16
Initial particle images (no.)	246,755	183,241
Final particle images (no.)	41,693	24,936
Map resolution (Å)	3.5	5
FSC threshold	0.143	0.143
Map resolution range (Å)	3.3 -5.5	4.1 -9.2
**Refinement**		
Initial model used (PDB code)	3NSJ	
Model resolution (Å)		
FSC threshold	0.5	
Model resolution range (Å)	3.8	
Map sharpening *B* factor (Å^2)	-104.6	
Model composition		
Non-hydrogen atoms	75712	
Protein residues	9664	
Ligands	32	
B factors (Å^2)		
Protein	36.62	
Ligand	40.47	
R.m.s. deviations		
Bond lengths (Å)	0.002	
Bond angles (°)	0.552	
Validation		
MolProbity score	1.97	
Clashscore	13.12	
Poor rotamers (%)	0	
Ramachandran plot		
Favored (%)	95.10	
Allowed (%)	4.90	
Disallowed (%)	0	

Table S2. X-ray crystallographic statistics of P2 domain.

	Native 1 (2 copies)	Au-derivative	Native 2 (8 copies)**
**Data collection**			
Space group	$P2_1$	$P2_1$	P1
Cell dimensions			
a, *b*, *c* (Å)	70.53, 31.27, 103.65	71.01, 30.91, 103.31	70.23, 70.05, 128.14
α, β, γ (°)	90, 90.42, 90	90, 91.61, 90	78.5, 79.05, 85.01
Resolution (Å)	58.98 – 2.05 (2.09 -2.05)*	57.44 – 2.44 (2.48 – 2.44)	51.18 – 3.17 (3.28 – 3.17)
R_{merge}	0.07 (1.374)	0.126 (1.462)	0.2618 (1.858)
R_{pim}	0.03 (0.575)	0.054 (0.858)	0.0769 (0.3183)
$I / \sigma I$	12.7 (1.3)	9.9 (1.3)	10.41 (1.18)
Completeness (%)	99.4 (99.2)	99.7 (99.8)	95.30 (59.72)
Redundancy	6.5 (6.7)	6.6 (6.6)	10.7 (1.5)
CC-half	0.999 (0.661)	0.997 (0.545)	0.993 (0.778)
**Refinement**			
Resolution (Å)	51.81 – 2.05 (1.15 – 1.11)		51.18 – 3.17 (3.28 – 3.17)
No. reflections	189385 (33178)		405935 (3449)
Unique reflections	28963 (7980)		37940 (2362)
R_{work} / R_{free}	21.21% / 24.49%		18.87% / 22.46%
No. atoms			
Protein	2988		11992
Ligand/ion	110		
B-factors			
Protein	74.24		90.0
Ligand/ion	79.31		
R.m.s. deviations			
Bond lengths (Å)	0.009		0.003
Bond angles (°)	1.1		0.76

*Values in parentheses are for highest-resolution shell.

** Several datasets from the same crystal were merged together.

Movie S1. Pre-pore PFN2 oligomers on mica. High-speed atomic force microscopy (HS-AFM) image sequence of perforin-2 pre-pore assembly dynamics on mica.

Movie S2. Pre-pore PFN2 oligomers on a supported lipid bilayer. High-speed atomic force microscopy (HS-AFM) image sequence of hexagonal-packed perforin-2 pre-pores freely formed on a supported lipid bilayer.

Movie S3. Mobility of membrane-bound pre-pore PFN2 oligomers. High-speed atomic force microscopy (HS-AFM) movie of a force-sweep experiment in which the tip of the HS-AFM is used to sweep membrane-bound pre-pores from the field of view, and they are allowed to redistribute themselves when the tip force is subsequently minimized.

Movie S4. Real-time pore formation imaged by HS-AFM. High-speed atomic force microscopy (HS-AFM) experiment showing the conformational change occurring when membrane-bound pre-pore PFN2 oligomers are exposed to an acid shock (see Materials and Methods for more details). The pre-pore to pore transition starts at 14 seconds after injection of 0.1M HCl and results in a ~4nm increase in the oligomer height above the surface to which it is attached, and the transition finishes within 3 seconds. The break up of many ring form pre-pores into arcs is probably due to the sudden change in pH caused by addition of 0.1M HCl. See also Main Text Fig. 5.
